# Supplementary figures and images for: Mediator complex subunit MED23 dampens antiviral innate immunity by restricting RIG-I expression
Source: PLoS Biol. 2025 Jul 24;23(7):e3003294. doi: 10.1371/journal.pbio.3003294 (PMC12316392; doi:10.1371/journal.pbio.3003294)

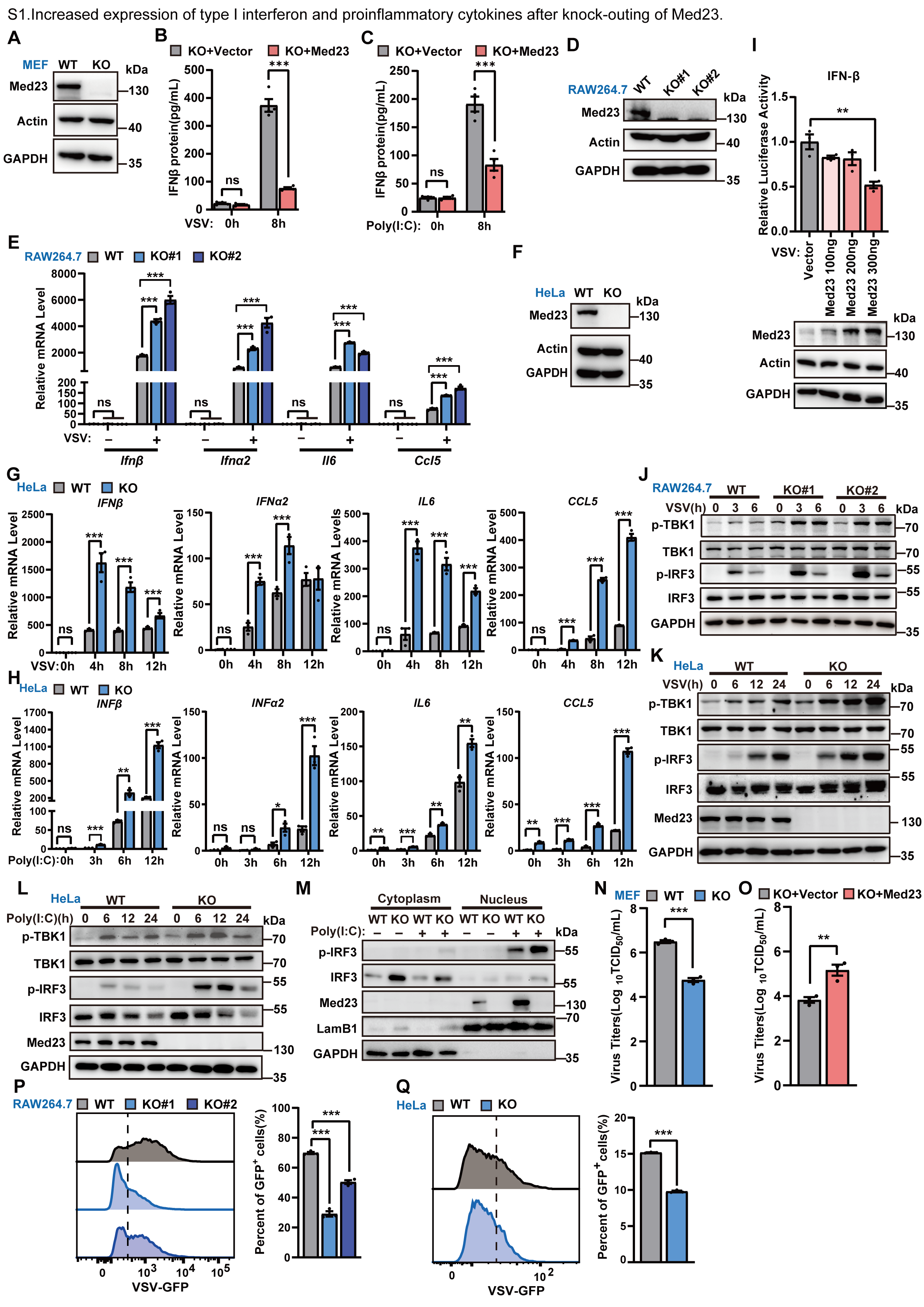

Supplement: S1 Fig — (A) Immunoblotting to detect Med23 in wild-type and Med23-knockout MEF cells. (B) and (C) ELISA analysis of IFNβ in the supernatant of Med23-overexpressing KO MEFs (n = 4) infected with VSV (MOI = 1) or stimulated with poly(I:C) (5 μg/mL) for 8 h. (D) Immunoblotting was used to detect Med23 in wild-type and Med23-knockout RAW264.7 cells. (E) qPCR analysis of the levels of the indicated genes in Med23 knockout RAW264.7 cells infected with VSV (MOI = 1) for 6 h. (F) Immunoblotting to detect Med23 in wild-type and Med23-knockout HeLa cells. (G) and (H) qPCR analysis of the levels of the indicated genes in Med23-knockout HeLa cells (n = 3) infected with VSV (MOI = 1) or stimulated with poly(I:C) (5 μg/mL) for the indicated times. (I) Med23 knockout HeLa cells (n = 3) were transiently transfected with IFNβ reporter plasmids along with Med23-expressing plasmids (0, 100, 200, and 300 ng). After 48 h, a luciferase assay was performed in which the cells were stimulated with VSV (MOI = 1) for 8 h. The results are presented relative to the luciferase activity in control cells (transfected with the luciferase reporter and empty vector with stimulation of VSV). The results of the immunoblot analysis of Med23 are shown below. Actin and GAPDH served as loading controls. (J) Immunoblot analysis of the indicated protein in Med23 knockout RAW264.7 cells infected with VSV (MOI = 1). (K) and (L) Immunoblot analysis of the indicated protein in Med23-knockout HeLa cells infected with VSV (MOI = 1) or stimulated with poly(I:C) (5 μg/mL). (M) Immunoblot analysis of p-IRF3 protein in the nuclear and cytoplasmic fractions of WT and Med23-knockout HeLa cells upon stimulation with poly(I:C) (5 μg/mL) for 8 h. GAPDH served as a cytoplasmic control. Lamin B1 served as a nuclear protein control. (N) TCID50 assay of virus titers of WT and Med23 KO MEFs (n = 3) after VSV infection (MOI = 1). (O) TCID50 assay of virus titers of Med23-overexpressing KO MEFs (n = 3) after VSV infection (MOI = 1) for 8 [file pbio.3003294.s001.tif]

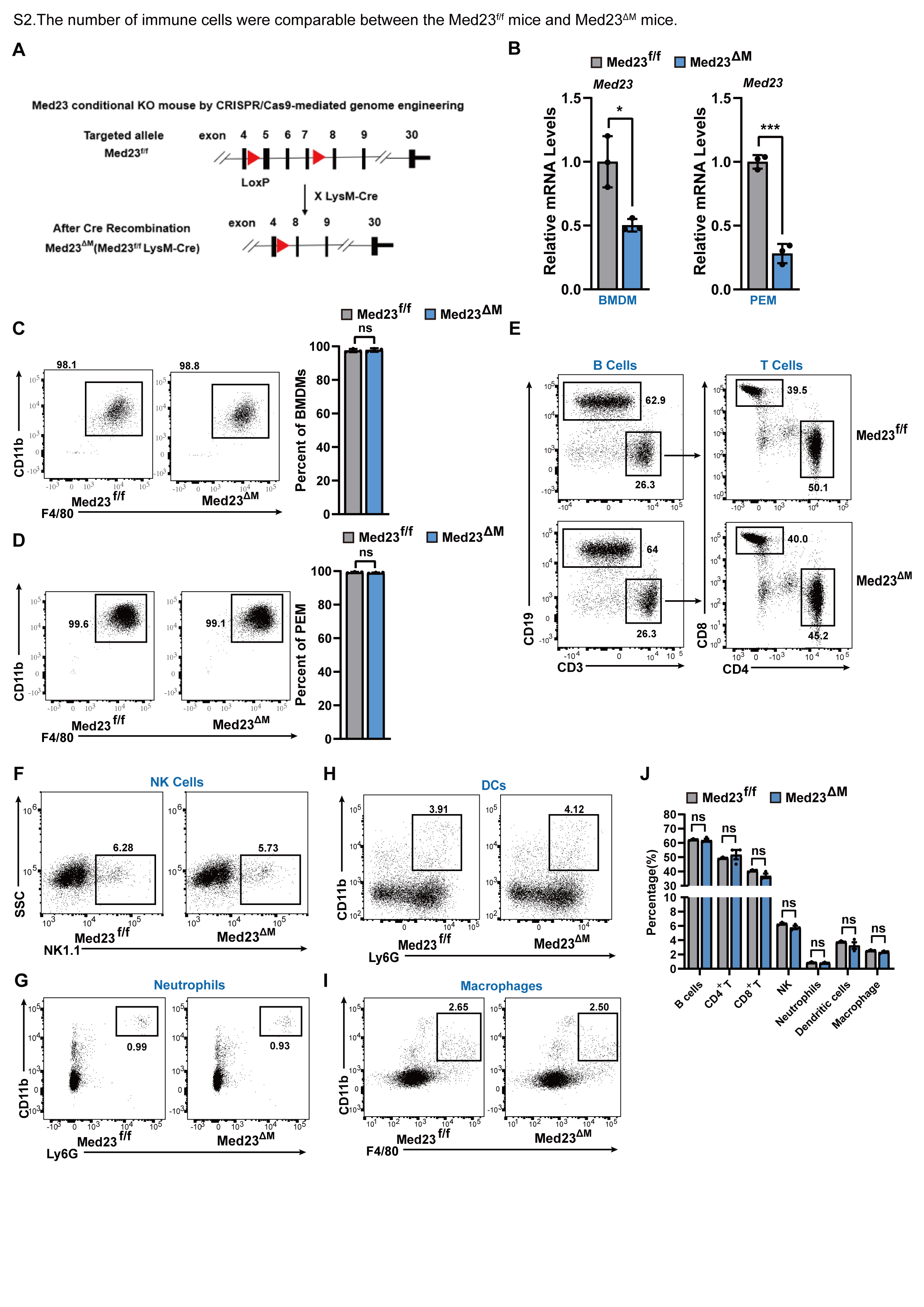

Supplement: S2 Fig — (A) Strategy for generation of macrophage-specific Med23 conditional knockout mice. (B) qPCR analyses of Med23 gene expression in BMDMs and PMs from Med23f/f and Med23ΔM mice (n = 3). (C) and (D) Flow cytometric analysis of the percentages of BMDMs or PEMs from Med23f/f and Med23ΔM mice (n = 3). (E–I) Flow cytometric analysis of B cells, CD4+ T cells, CD8+ T cells, NK cells, neutrophils, dendritic cells and macrophages in the spleens of Med23f/f and Med23ΔM mice (n = 3). (J) Statistical analysis of the percentages of immune cells in E–I. Data present means ± SEM of three independent experiments. Statistical analysis in (B–C), and (J) was performed by two-tailed unpaired Student t test; ns, not significant. The data underlying this figure can be found in S2 Data. (TIF) [file pbio.3003294.s002.tif]

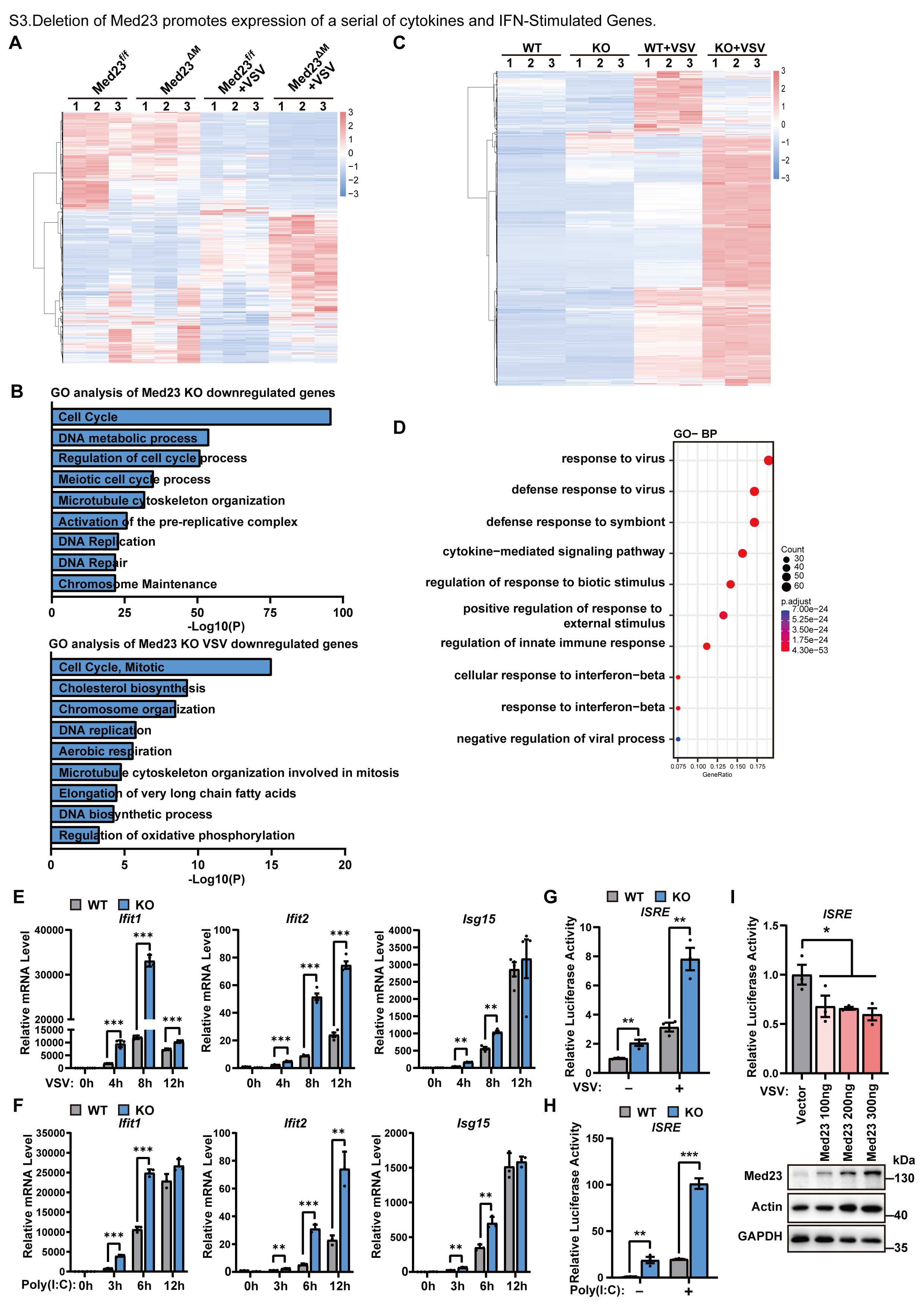

Supplement: S3 Fig — (A) Cluster heatmap of the RNA-seq data showing the differentially expressed genes in the Med23f/f and Med23ΔM BMDMs without or with VSV infection. (B) Gene ontology analysis of down-regulated genes the Med23f/f and Med23ΔM BMDMs without or with VSV infection according to RNA-seq data. (C) Cluster heatmap of the RNA-seq data showing the differentially expressed genes in the wild-type and Med23-knockout MEFs without or with VSV infection. (D) Gene ontology analysis of increased genes in WT and Med23 knockout MEFs infected with VSV according to RNA-seq data. (E) and (F) qRT-PCR analysis of ISGs in WT and Med23 knockout MEFs infected with VSV (n = 4) or stimulated with poly(I:C) (n = 3) for the indicated times. (G) and (H) Luciferase reporter assays were conducted with WT and Med23-knockout HeLa cells (n = 3) transfected for 48 h with ISRE reporter plasmids and then infected with VSV (MOI = 1) or stimulated with poly(I:C) (5 μg/mL) for 8 h. (I) Med23 knockout HeLa cells (n = 3) were transiently transfected with IFNβ reporter plasmids along with Med23-expressing plasmids (0, 100, 200, and 300 ng). After 48 h, a luciferase assay was performed in which the cells were stimulated with VSV (MOI = 1) for 8 h. The results are presented relative to the luciferase activity in control cells (transfected with the luciferase reporter and empty vector with stimulation of VSV). The results of the immunoblot analysis of Med23 are shown below. Data present means ± SEM of at least three independent experiments. Statistical analysis in (E–I) was performed by two-tailed unpaired Student’s t test; *P < 0.05, **P < 0.01, and ***P < 0.001. The data underlying this figure can be found in S2 Data. (TIF) [file pbio.3003294.s003.tif]

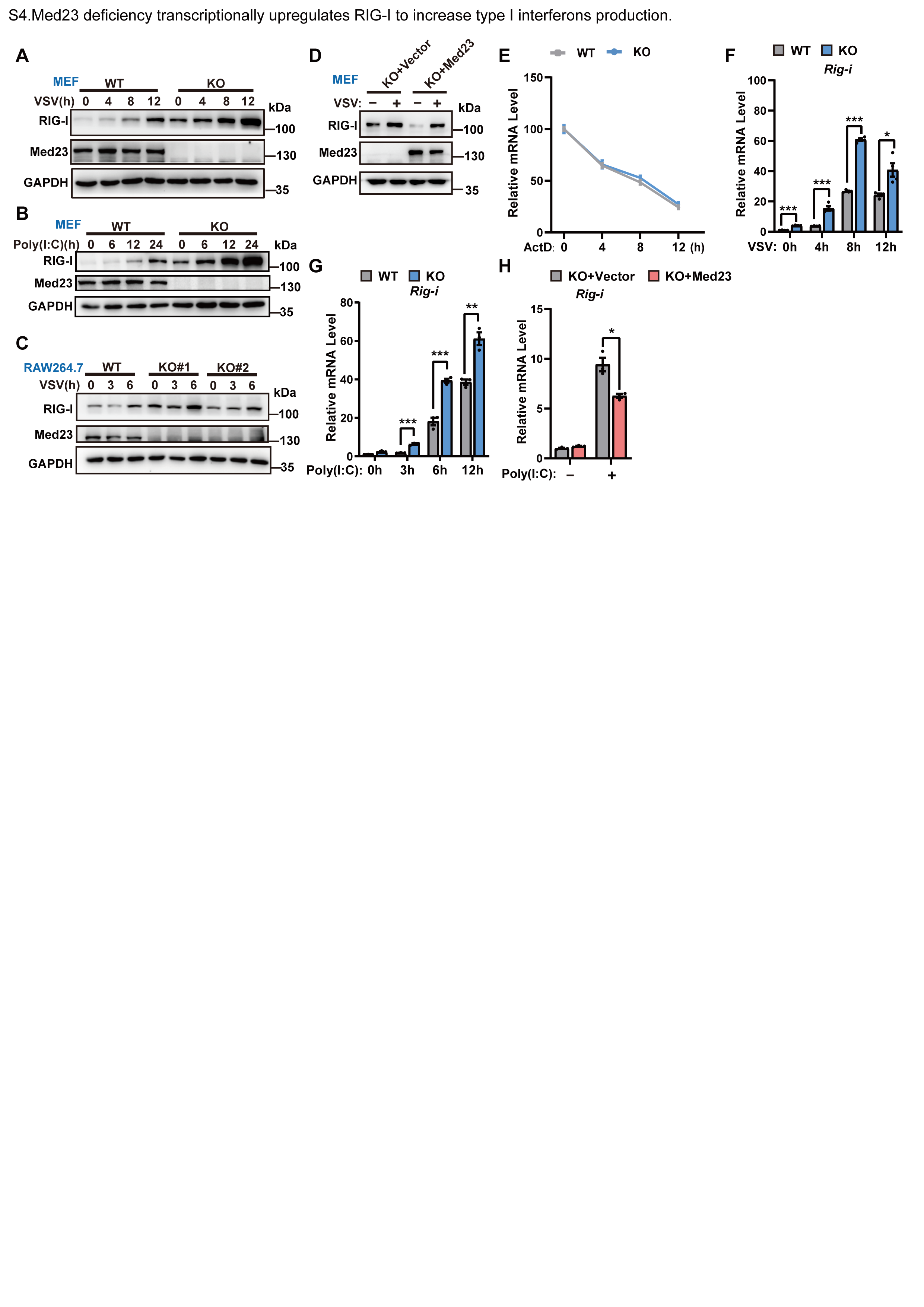

Supplement: S4 Fig — (A) and (B) Immunoblot analysis of RIG-I in WT and Med23 knockout MEFs (n = 3) infected with VSV (MOI = 1) or stimulated with poly(I:C) (5 µg/mL) for the indicated times. (C) Immunoblot analysis of RIG-I in WT and Med23 knockout RAW264.7 cells infected with VSV (MOI = 0.5) for the indicated times. (D) Immunoblot analysis of RIG-I protein expression in Med23-overexpressing knockout MEFs infected with VSV (MOI = 1) for 8 h. (E) qPCR analysis of RIG-I in WT and Med23 knockout HeLa cells (n = 3) treated with Act D (1 mM/mL) for the indicated times. (F) and (G) qPCR analysis of RIG-I mRNA levels in WT and Med23 knockout MEFs infected with VSV (n = 4, MOI = 1) or stimulated with poly(I:C) (n = 3, 5 µg/mL) for the indicated times. (H) qPCR analysis of RIG-I mRNA levels in Med23-overexpressing knockout MEFs (n = 3) stimulated with poly(I:C) (5 µg/mL) for 8 h. Data present means ± SEM of at least three independent experiments. Statistical analysis in (F–H) was performed by two-tailed unpaired Student t test; *P < 0.05, **P < 0.01, and ***P < 0.001. The data underlying this figure can be found in S2 Data. (TIF) [file pbio.3003294.s004.tif]

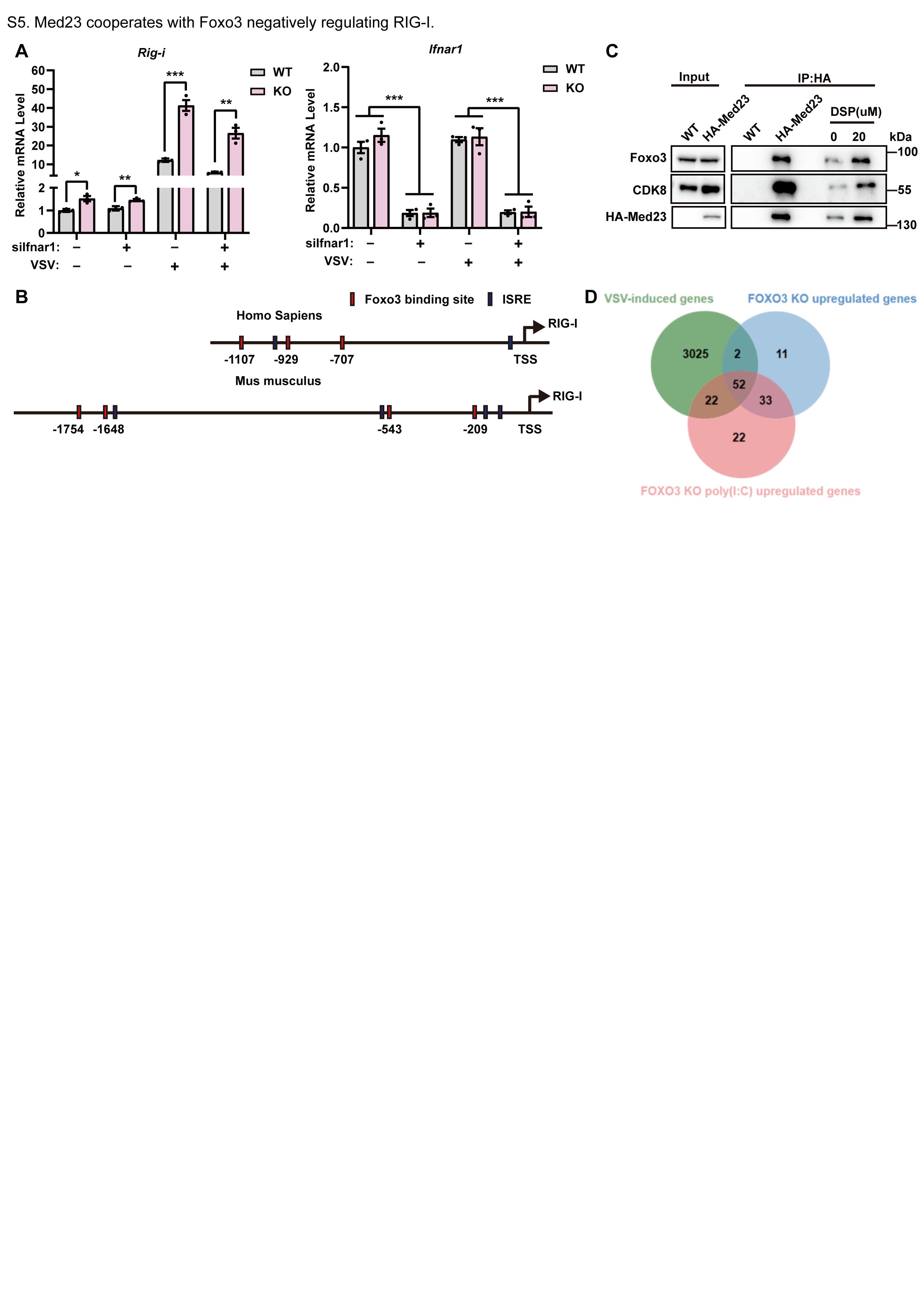

Supplement: S5 Fig — (A) qPCR analysis of Rig-i and Ifnar1 mRNAs in WT and Med23 knockout MEFs (n = 3) with Ifnar1 knockdown (siRNA = 50nM) infected with VSV (MOI = 1) for 8 h. (B) schematic illustration of the predicted binding sites of Foxo3 on the RIG-I promoter was generated utilizing the resource provided at https://epd.epfl.ch. (C) Immunoblot analysis of endogenous Foxo3 in the cell lysates of HA-Med23-KI HEK293T cells treated with DSP (20uM) immunoprecipitated with the HA antibody. (D) Venn diagram to show the overlap of these genes upregulated in FOXO3 KO cells treated with or without poly(I:C) and those induced by VSV infection in our RNA-seq analysis. Data present means ± SEM of three independent experiments. Statistical analysis in (A) was performed by two-tailed unpaired Student t test; *P < 0.05, **P < 0.01, and ***P < 0.001. The data underlying this figure can be found in S2 Data. (TIF) [file pbio.3003294.s005.tif]
