# Supplementary material for: Mediator complex subunit MED23 dampens antiviral innate immunity by restricting RIG-I expression
Source: PLoS Biol. 2025 Jul 24;23(7):e3003294. doi: 10.1371/journal.pbio.3003294 (PMC12316392; doi:10.1371/journal.pbio.3003294)
Supplement: S1 Table — (PDF) [file pbio.3003294.s006.pdf]

Table S1. Primer sequences used in the qRT-PCR analysis

Primer name	Sequence (5’-3’)
Mus-Med23-F	TCGGAAAATCATTGGAGGAG
Mus-Med23-R	CAATAGGCAGGCATTTCGTT
Mus-Ifnβ-F	GCACTGGGTGGAATGAGACTATTG
Mus-Ifnβ-R	TTCTGAGGCATCAACTGACAGGTC
Mus-Ifnα-F	TGACCTGCAAACCTGTCTGA
Mus-Ifnα-R	ACTTCTGCTCTGACCACCTC
Mus-Il6-F	AGTTGCCTTCTTGGGACTGA
Mus-Il6-R	TCCACGATTTCCCAGAGAAC
Mus-Ccl5-F	GCTGCTTTGCCTACCTCTCC
Mus-Ccl5-R	TCGAGTGACAAACACGACTGC
Mus-Ifit1-F	CTGAGATGTCACTTCACATGGAA
Mus-Ifit1-R	GTGCATCCCCAATGGGTTCT
Mus-Ifit2-F	AGTACAACGAGTAAGGAGTCACT
Mus-Ifit2-R	AGGCCAGTATGTTGCACATGG
Mus-Isg15-F	GGTGTCCGTGACTAACTCCAT
Mus-Isg15-R	TGGAAAGGGTAAGACCGTCCT
VSV-G-F	TGATAGTACCGGAGGATTGACGAC
VSV-G-R	CCTTGCAGTGACATGACTGCTCTT
Mus-Rig-i-F	AAGAGCCAGAGTGTCAGAATCT
Mus-Rig-i-R	AGCTCCAGTTGGTAATTTCTTGG
Mus-Gapdh-F	AGGTCGGTGTGAACGGATTTG
Mus-Gapdh-R	TGTAGACCATGTAGTTGAGGTCA
Homo-MED23-F	AGGACCAGTTGGTGGATCTG
Homo-MED23-R	TCGCCCTGCTAACTTCTGAT
Homo-IFNβ-F	CAGCAATTTTCAGTGTCAGAAGCT
Homo-IFNβ-R	TCATCCTGTCCTTGAGGCAGTAT
Homo-IFNα-F	GACTTGTCTGCTACTTGGAATGC
Homo-IFNα-R	TTGGTTGAGGAAGAGGGCT
Homo-IL6-F	ACACACACACACACACACAC
Homo-IL6-R	TCCAGGCAAACGAGGTCTAG
Homo-CCL5-F	GAGGCTTCCCCTCACTATCC
Homo-CCL5-R	CTCAAGTGATCCACCCACCT

Homo-GAPDH-F	CGACCACTTTGTCAAGCTCA
Homo-GAPDH-R	AGGGGAGATTCAGTGTGGTG

F: Forward Primer R: Reverse Primer
