# Supplementary material for: Mediator complex subunit MED23 dampens antiviral innate immunity by restricting RIG-I expression
Source: PLoS Biol. 2025 Jul 24;23(7):e3003294. doi: 10.1371/journal.pbio.3003294 (PMC12316392; doi:10.1371/journal.pbio.3003294)
Supplement: S2 Table — (PDF) [file pbio.3003294.s007.pdf]

**Table S2. Primer sequences used in the ChIP-qPCR analysis（For Pol II）**

| Primer name | Sequence (5'-3') |
|---|---|
| Mus-RIG-I-ChIP-A-F | TGGGGGCAGGGGTAAGAAAG |
| Mus-RIG-I-ChIP-A-R | TTCATCTCCTCTGTCGCCAT |
| Mus-RIG-I-ChIP-B-F | CCTTTGAACACTTGGGCTCC |
| Mus-RIG-I-ChIP-B-R | CAGCCACATTGCATCTCCTC |
| Mus-RIG-I-ChIP-C-F | CTCACAGGTTGCCATCCATG |
| Mus-RIG-I-ChIP-C-R | CCTTACTGCACAATGAGGCC |
| Mus-RIG-I-ChIP-D-F | GCCCTTGGTCACAGAGATCT |
| Mus-RIG-I-ChIP-D-R | AGCACTGTTCCTTCCCTGAA |

F: Forward Primer R: Reverse Primer

**Primer sequences used in the ChIP-qPCR analysis（For Foxo3）**

| Primer name | Sequence (5'-3') |
|---|---|
| Mus-RIG-I-ChIP-A-F | GAAATATAAAGCCACCACGCG |
| Mus-RIG-I-ChIP-A-R | CTGGCCATAGGAAATCGAA |
| Mus-RIG-I-ChIP-B-F | CACTTTACATCCCACTCACT |
| Mus-RIG-I-ChIP-B-R | CTTGATATGCCAGTGTGTG |
| Mus-RIG-I-ChIP-C-F | CTGCAGTAACCCAGGTGAAGT |
| Mus-RIG-I-ChIP-C-R | GACTTCTGATTGTATACTCTCA |
| Mus-RIG-I-ChIP-D-F | GACGTATAAAAGATTTGTACCC |
| Mus-RIG-I-ChIP-D-R | GGATGTTTATCAGGGCTGCG |

F: Forward Primer R: Reverse Primer
