# Supplementary material for: Mediator complex subunit MED23 dampens antiviral innate immunity by restricting RIG-I expression
Source: PLoS Biol. 2025 Jul 24;23(7):e3003294. doi: 10.1371/journal.pbio.3003294 (PMC12316392; doi:10.1371/journal.pbio.3003294)

A.Gating strategy for Fig.1M.

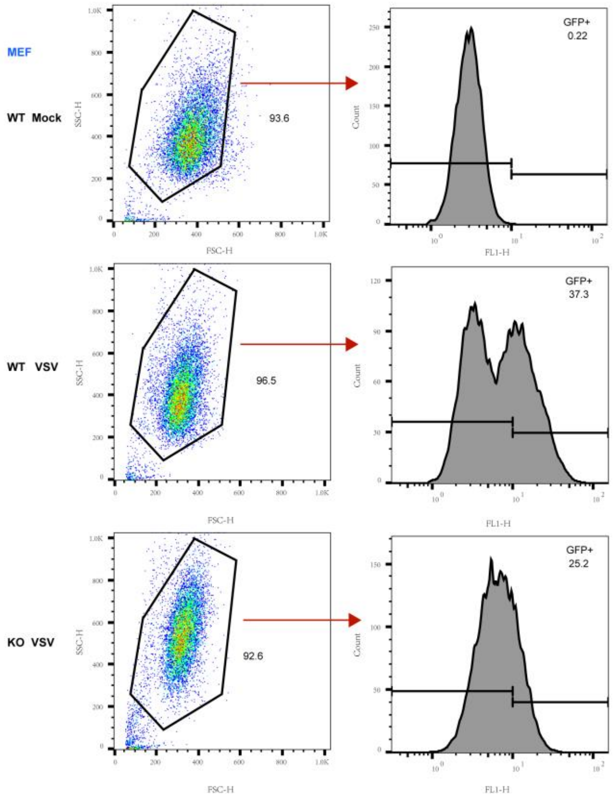

B.Gating strategy for Fig.1N.

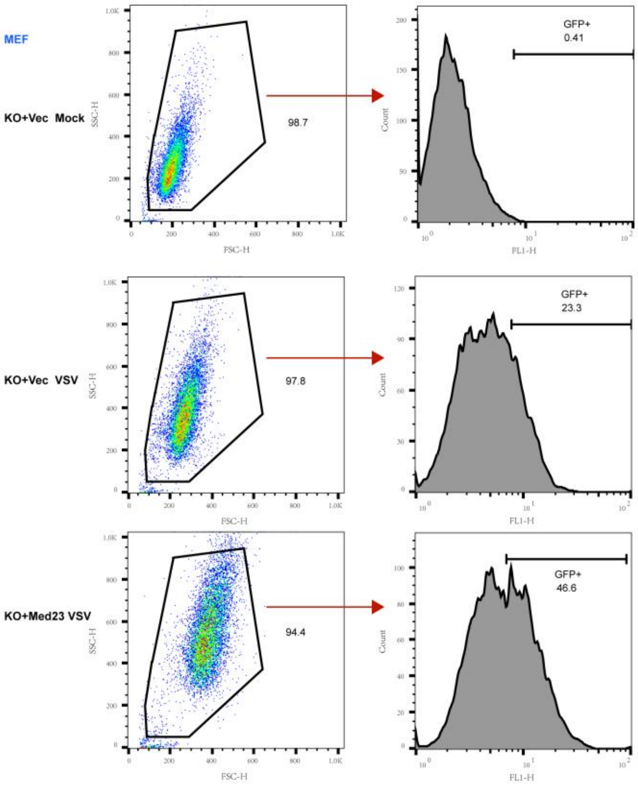

C.Gating strategy for Fig.S1P.

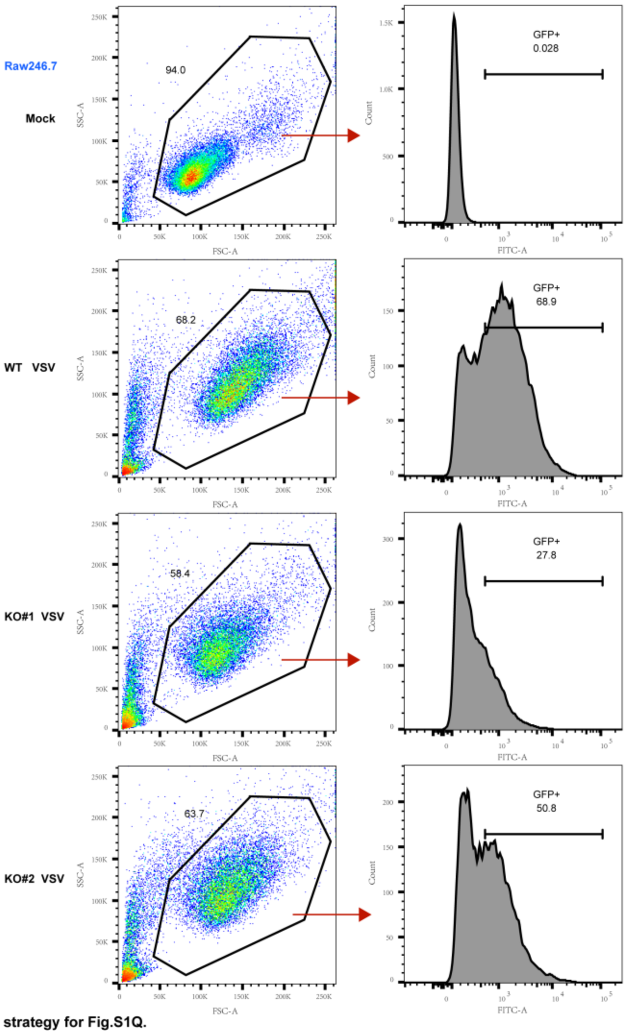

D.Gating strategy for Fig.S1Q.

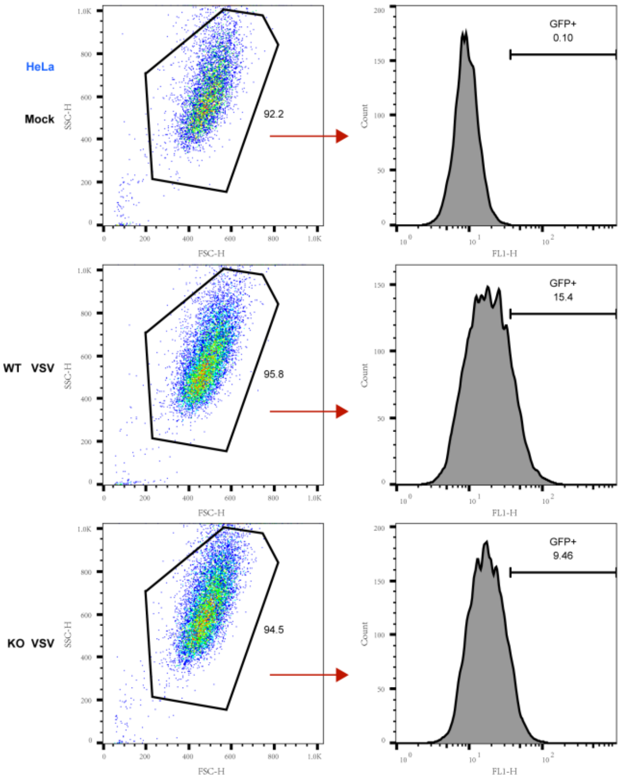

E.Gating strategy for Fig.S2C-D.

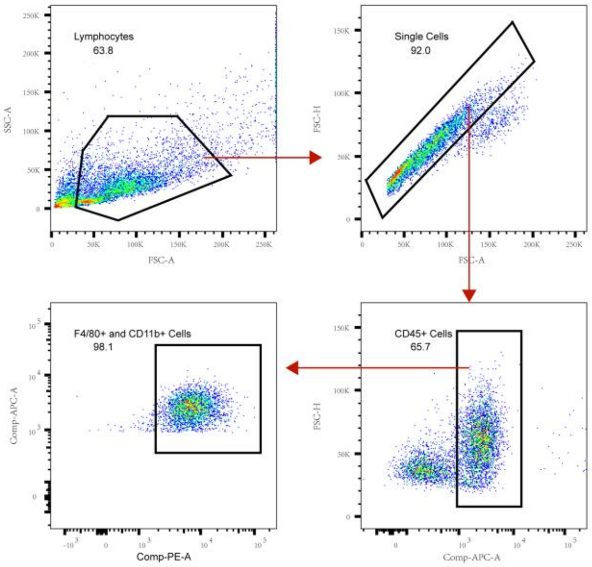

F.Gating strategy for Fig.S2E-F.

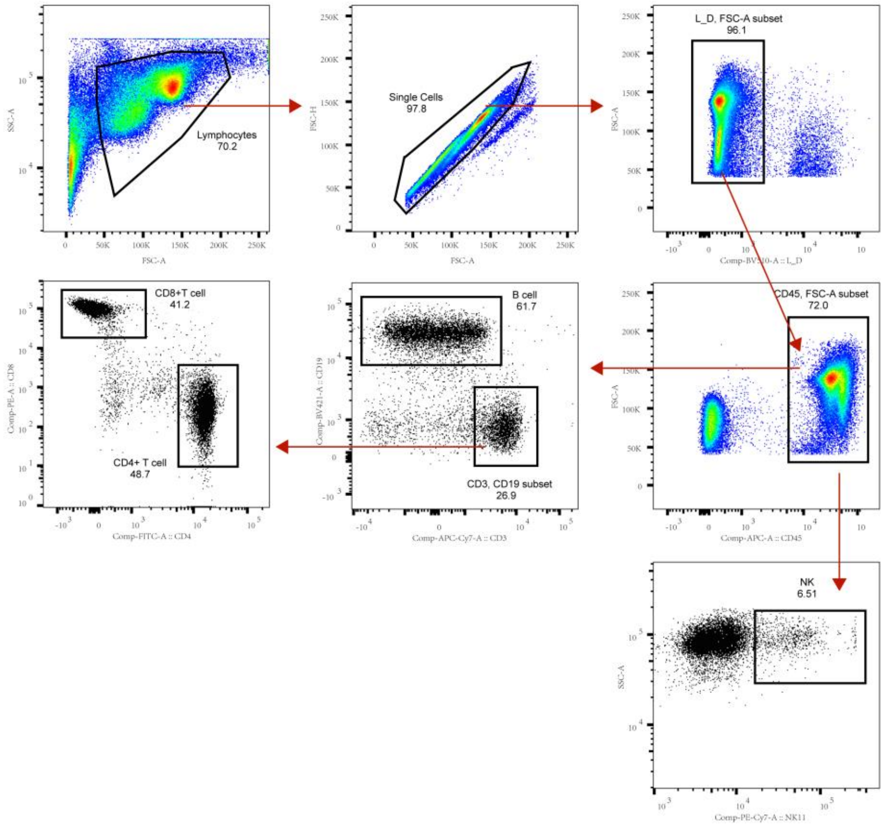

G.Gating strategy for Fig.S2G-I.

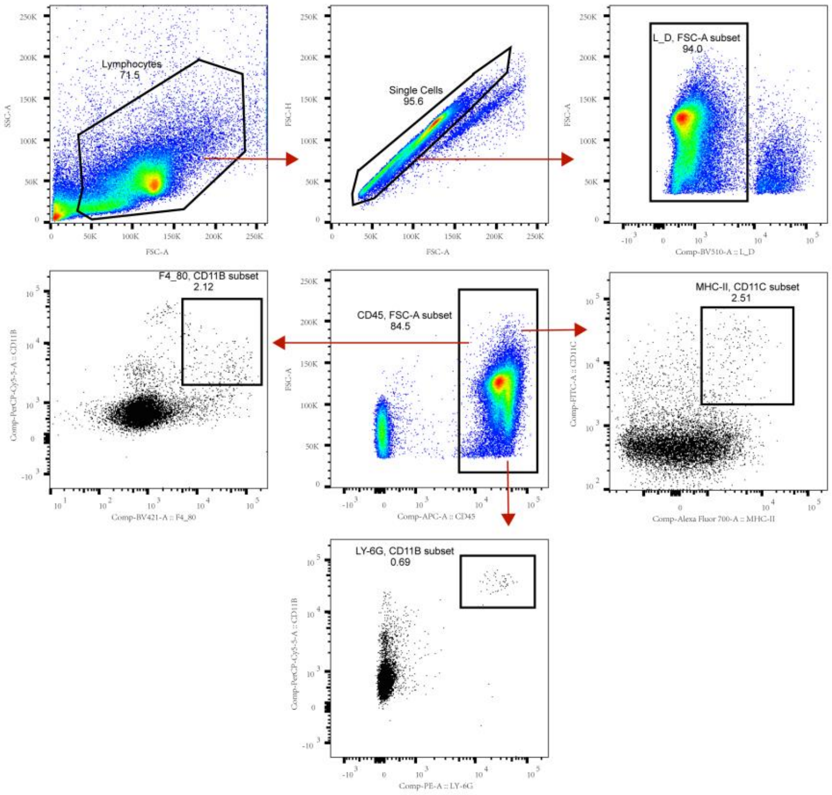

Supplement: S3 Data — (PDF) [file pbio.3003294.s010.pdf]
