# Supplementary material for: Mediator complex subunit MED23 dampens antiviral innate immunity by restricting RIG-I expression
Source: PLoS Biol. 2025 Jul 24;23(7):e3003294. doi: 10.1371/journal.pbio.3003294 (PMC12316392; doi:10.1371/journal.pbio.3003294)
Supplement: S1 Raw Images — (PDF) [file pbio.3003294.s011.pdf]

Fig.1E

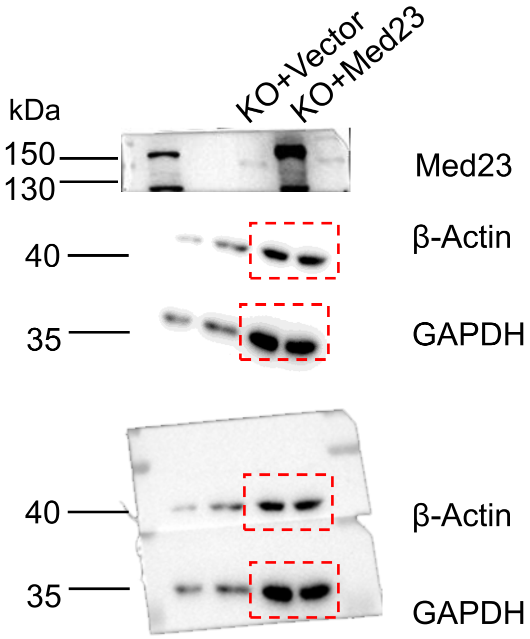
KO+Vector
KO+Med23
kDa
150
130
Med23
40
β-Actin
35
GAPDH
40
β-Actin
35
GAPDH

Fig.1J

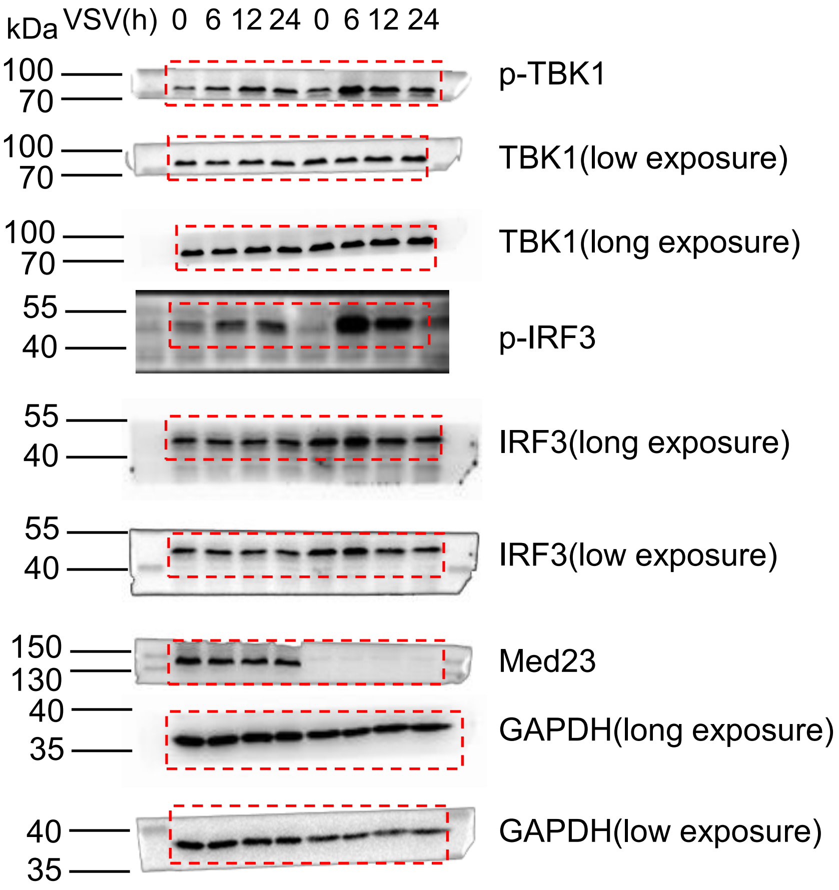

kDa
VSV(h) 0 6 12 24 0 6 12 24
100
70
p-TBK1
100
70
TBK1(low exposure)
100
70
TBK1(long exposure)
55
40
p-IRF3
55
40
IRF3(long exposure)
55
40
IRF3(low exposure)
150
130
Med23
40
35
GAPDH(long exposure)
40
35
GAPDH(low exposure)

Fig.1K

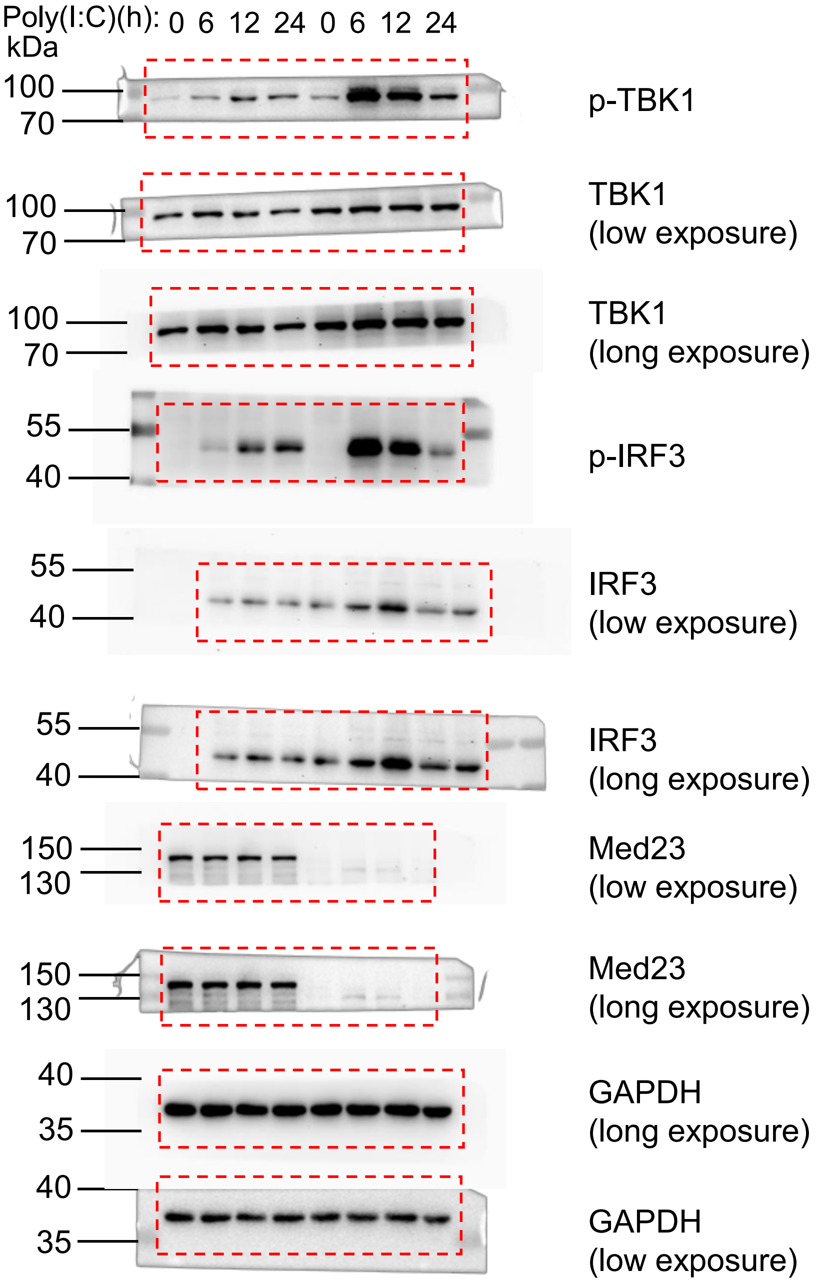
Poly(I:C)(h): 0 6 12 24 0 6 12 24
kDa
100
70
p-TBK1
100
70
TBK1
(low exposure)
100
70
TBK1
(long exposure)
55
40
p-IRF3
55
40
IRF3
(low exposure)
55
40
IRF3
(long exposure)
150
130
Med23
(low exposure)
150
130
Med23
(long exposure)
40
35
GAPDH
(long exposure)
40
35
GAPDH
(low exposure)

Fig.1L

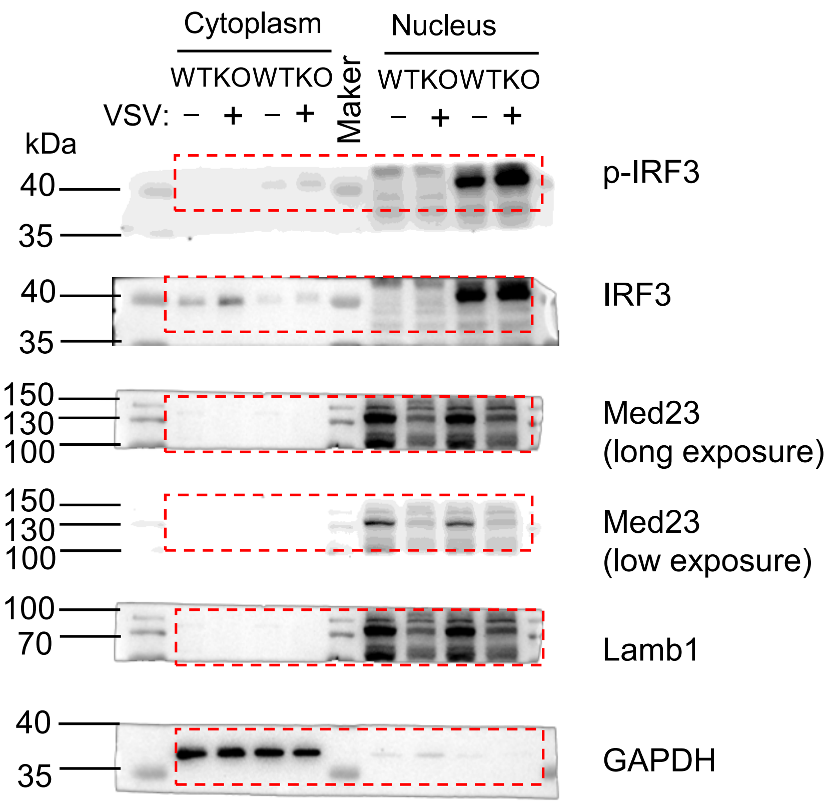
Cytoplasm
Nucleus
WT KO WT KO
Maker
WT KO WT KO
VSV: − + − +
− + − +
kDa
40
35
p-IRF3
40
35
IRF3
150
130
100
Med23
(long exposure)
150
130
100
Med23
(low exposure)
100
70
Lamb1
40
35
GAPDH

Fig.S1A

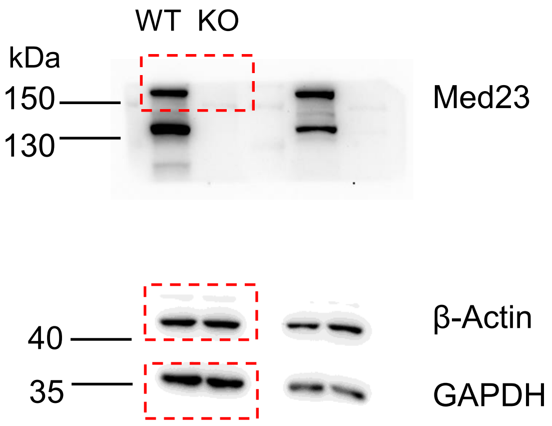

Fig.S1D

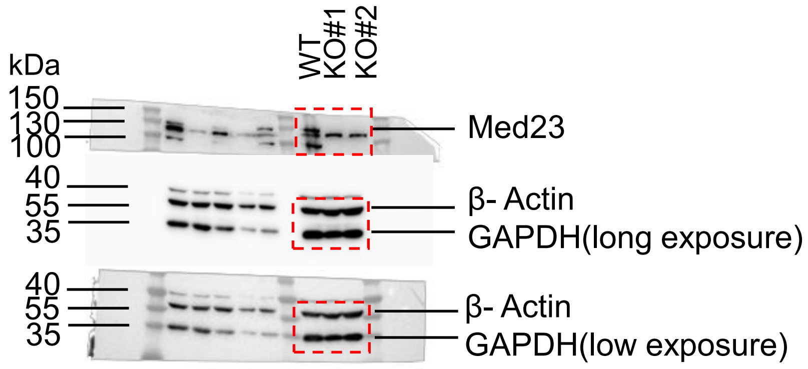
WT
KO#1
KO#2
kDa
150
130
100
Med23
40
55
35
β- Actin
GAPDH(long exposure)
40
55
35
β- Actin
GAPDH(low exposure)

Fig.S1F

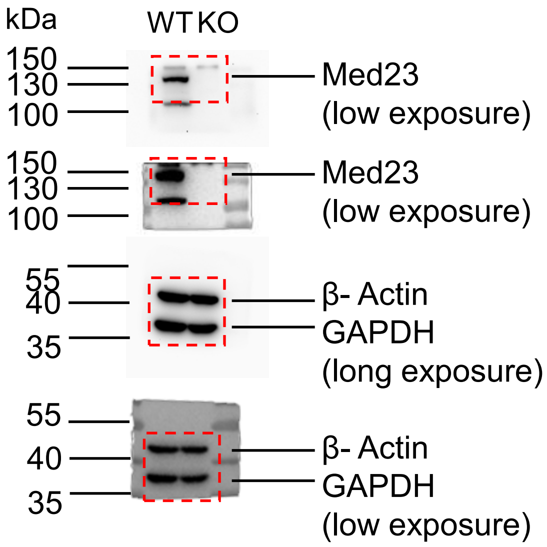
kDa
WT KO
150
130
100
Med23
(low exposure)
150
130
100
Med23
(low exposure)
55
40
35
β- Actin
GAPDH
(long exposure)
55
40
35
β- Actin
GAPDH
(low exposure)

Fig.S1I

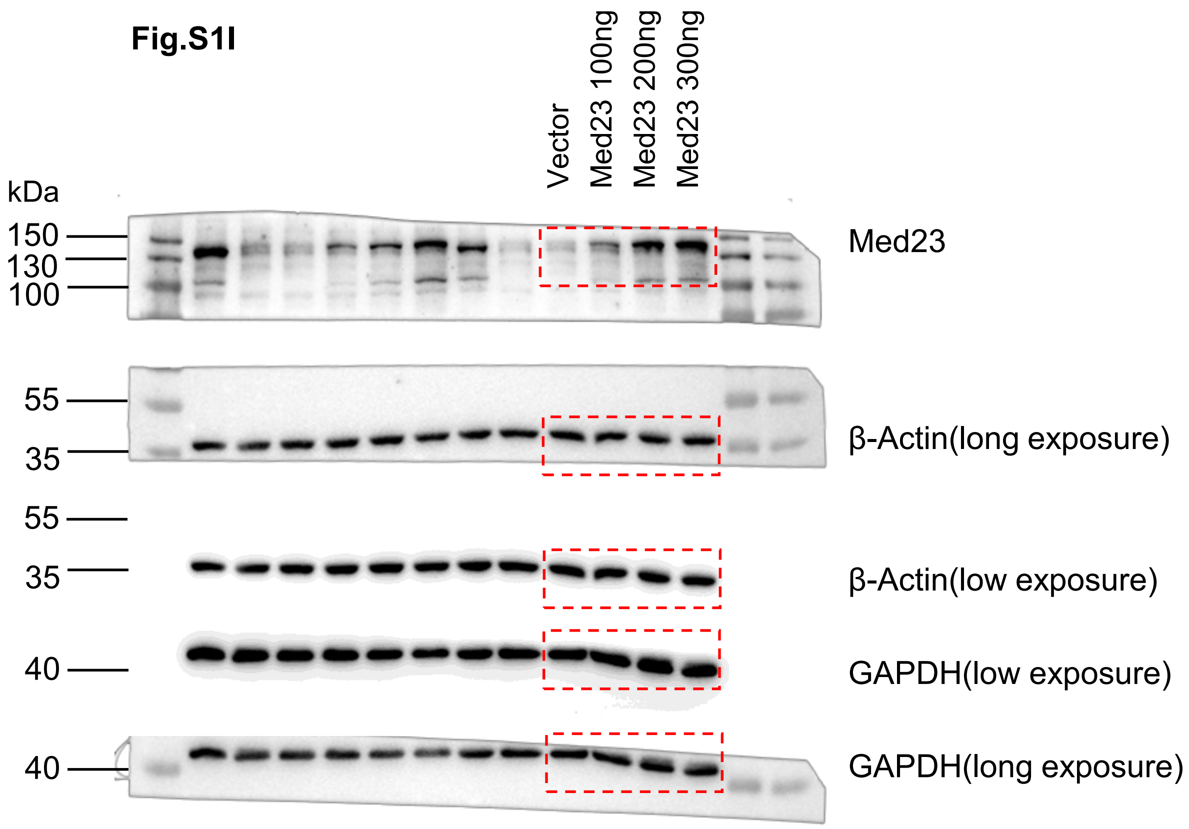
Vector
Med23 100ng
Med23 200ng
Med23 300ng
kDa
150
130
100
Med23
55
35
β-Actin(long exposure)
55
35
β-Actin(low exposure)
40
GAPDH(low exposure)
40
GAPDH(long exposure)

Fig.S1J

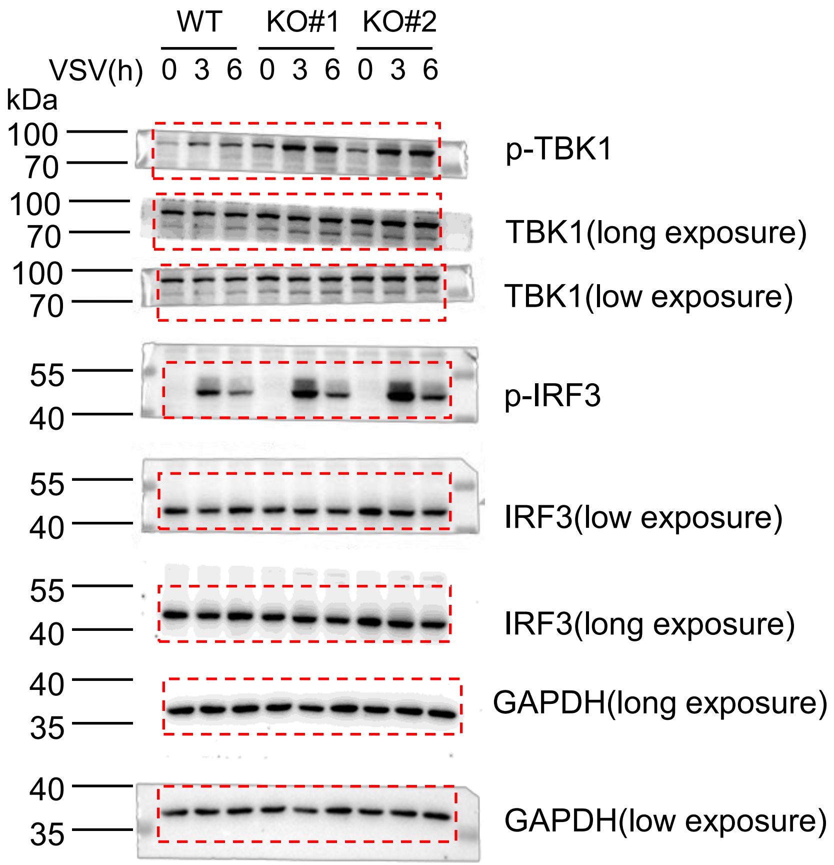
WT
KO#1
KO#2
VSV(h)
0 3 6 0 3 6 0 3 6
kDa
100
70
p-TBK1
100
70
TBK1(long exposure)
100
70
TBK1(low exposure)
55
40
p-IRF3
55
40
IRF3(low exposure)
55
40
IRF3(long exposure)
40
35
GAPDH(long exposure)
40
35
GAPDH(low exposure)

Fig.S1K

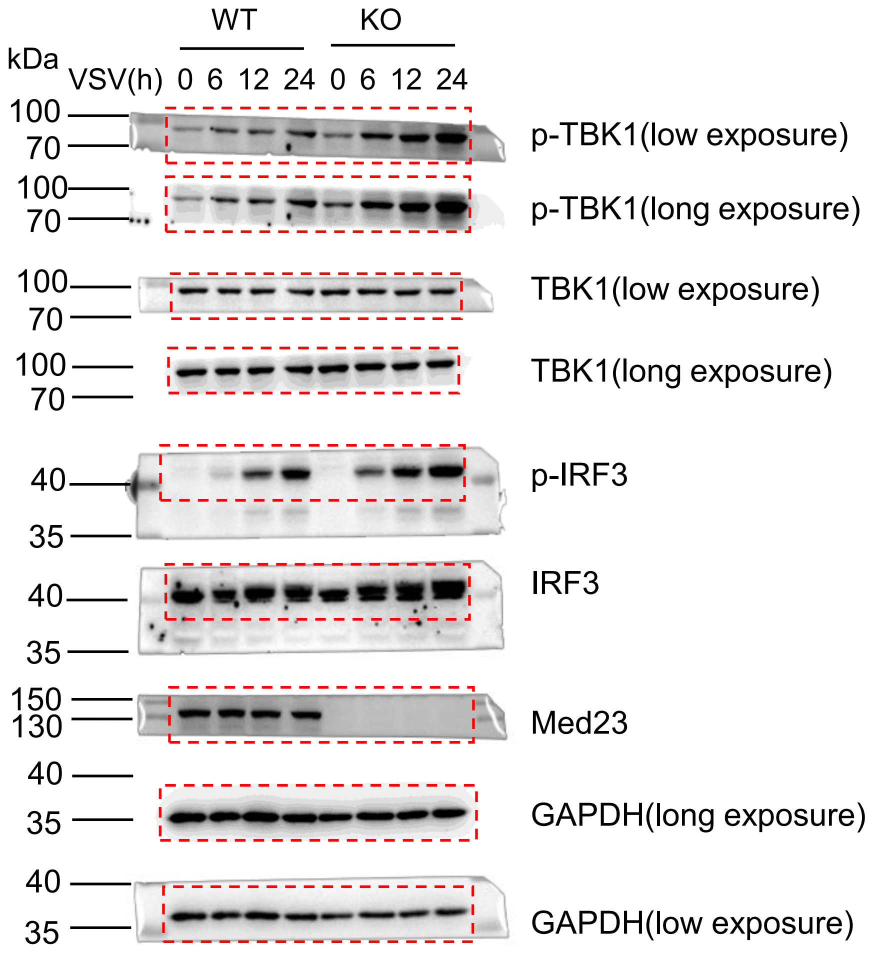
WT
KO
kDa
VSV(h) 0 6 12 24 0 6 12 24
100
70
p-TBK1(low exposure)
100
70
p-TBK1(long exposure)
100
70
TBK1(low exposure)
100
70
TBK1(long exposure)
40
35
p-IRF3
40
35
IRF3
150
130
Med23
40
35
GAPDH(long exposure)
40
35
GAPDH(low exposure)

Fig.S1L

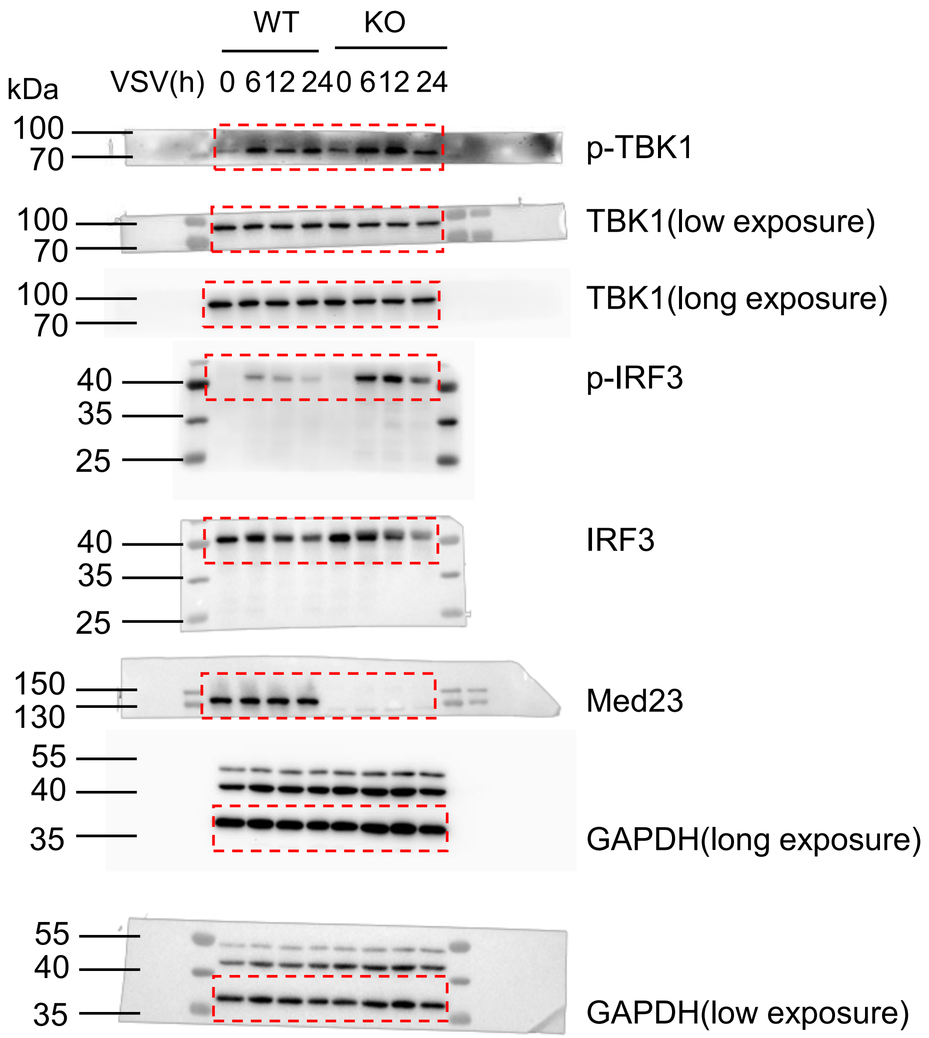

WT
KO
kDa
VSV(h) 0 6 12 24 0 6 12 24
100
70
p-TBK1
100
70
TBK1(low exposure)
100
70
TBK1(long exposure)
40
35
25
p-IRF3
40
35
25
IRF3
150
130
Med23
55
40
35
GAPDH(long exposure)
55
40
35
GAPDH(low exposure)

Fig.S1M

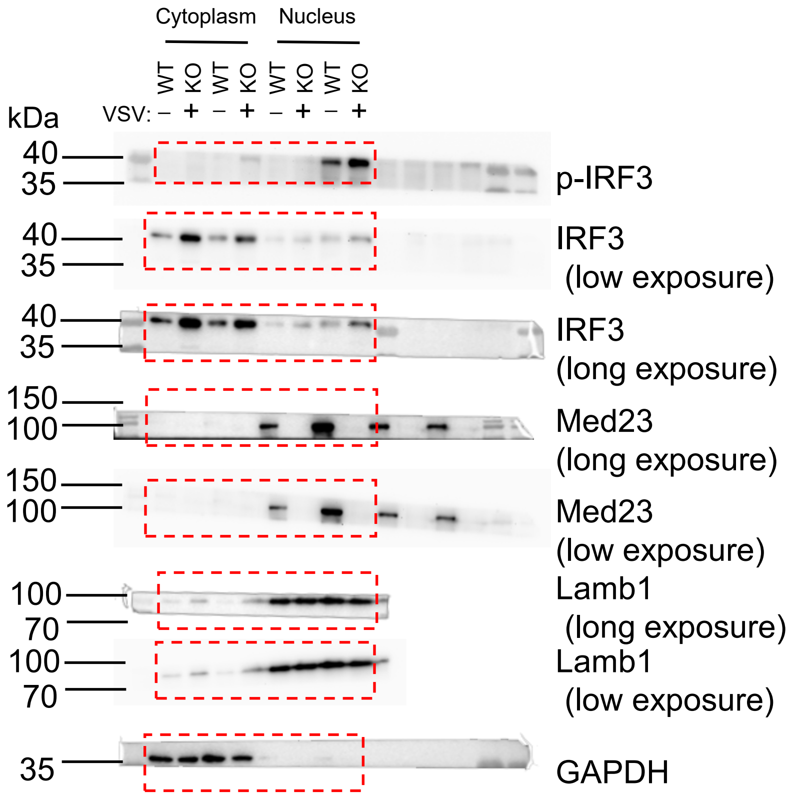

Cytoplasm
Nucleus
WT
KO
WT
KO
WT
KO
WT
KO
VSV: – + – + – + – +
kDa
40
35
p-IRF3
40
35
IRF3
(low exposure)
40
35
IRF3
(long exposure)
150
100
Med23
(long exposure)
150
100
Med23
(low exposure)
100
70
Lamb1
(long exposure)
100
70
Lamb1
(low exposure)
35
GAPDH

Fig.2A

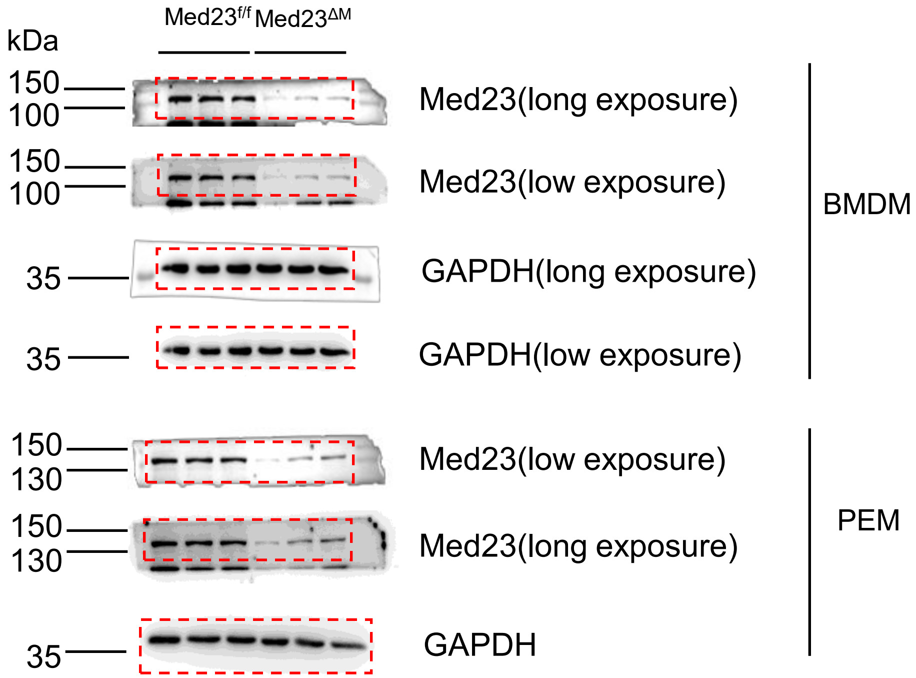
Med23f/f
Med23ΔM
kDa
150
100
Med23(long exposure)
150
100
Med23(low exposure)
35
GAPDH(long exposure)
35
GAPDH(low exposure)
BMDM
150
130
Med23(low exposure)
150
130
Med23(long exposure)
35
GAPDH
PEM

Fig.2D

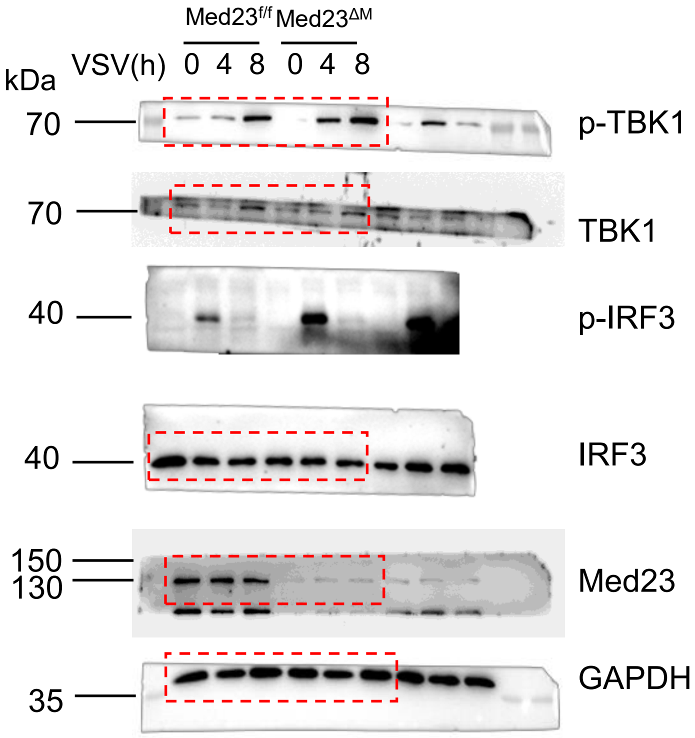
Med23$^{f/f}$
Med23$^{\Delta M}$
VSV(h)
0 4 8 0 4 8
kDa
70
p-TBK1
70
TBK1
40
p-IRF3
40
IRF3
150
130
Med23
35
GAPDH

Fig.2E

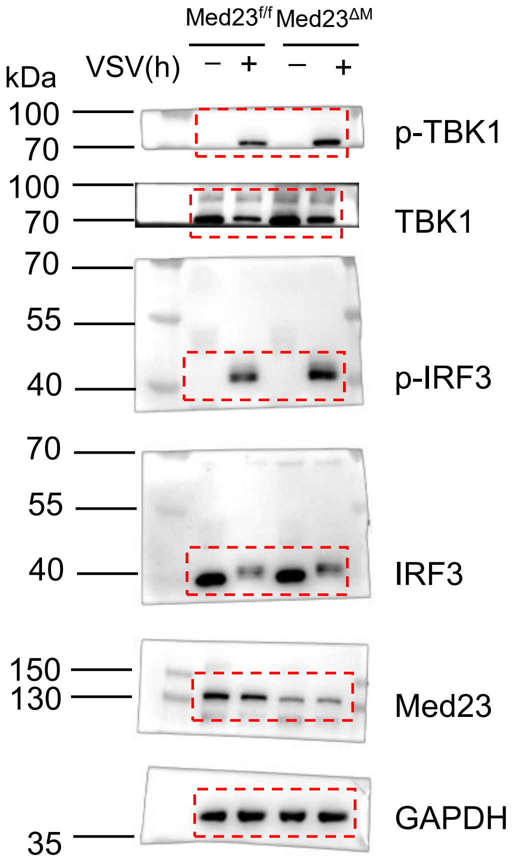

Med23f/f
Med23ΔM
kDa
VSV(h)
–
+
–
+
100
70
p-TBK1
100
70
TBK1
70
55
40
p-IRF3
70
55
40
IRF3
150
130
Med23
35
GAPDH

Fig.S3I

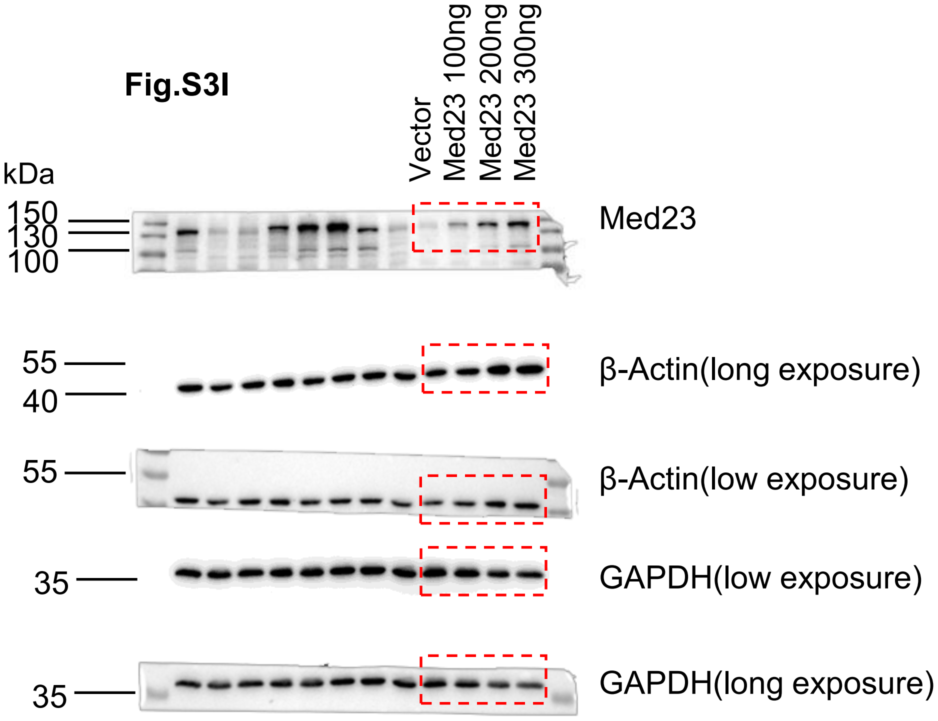
Vector
Med23 100ng
Med23 200ng
Med23 300ng
kDa
150
130
100
Med23
55
40
β-Actin(long exposure)
55
β-Actin(low exposure)
35
GAPDH(low exposure)
35
GAPDH(long exposure)

Fig.5B

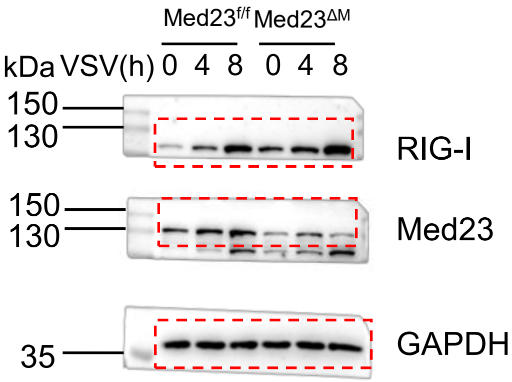
Med23f/f Med23ΔM
kDa VSV(h) 0 4 8 0 4 8
150
130
RIG-I
150
130
Med23
35
GAPDH

Fig.5D

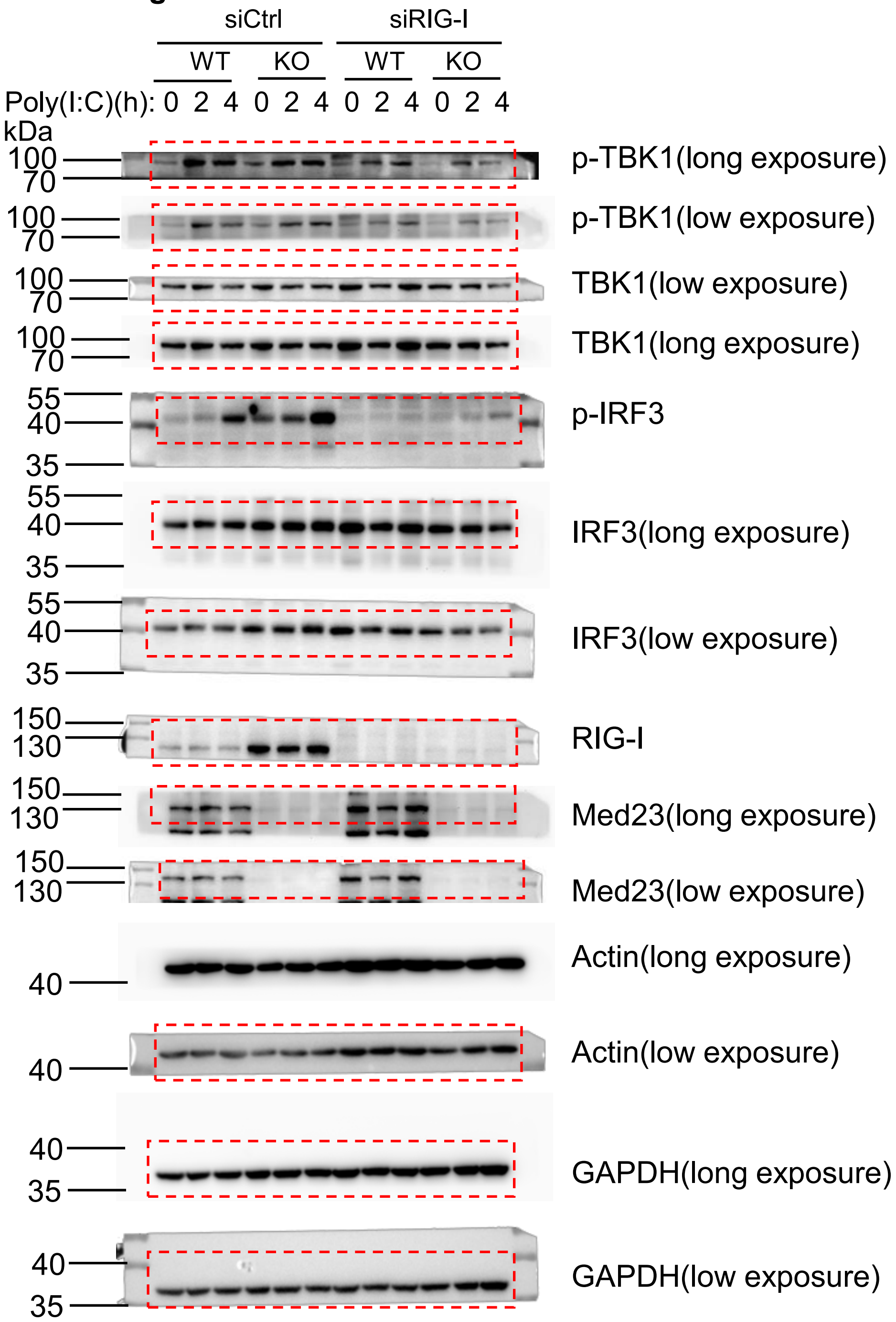
siCtrl
siRIG-I
WT
KO
WT
KO
Poly(I:C)(h): 0 2 4 0 2 4 0 2 4 0 2 4
kDa
100
70
p-TBK1(long exposure)
p-TBK1(low exposure)
TBK1(low exposure)
TBK1(long exposure)
55
40
35
p-IRF3
IRF3(long exposure)
IRF3(low exposure)
150
130
RIG-I
Med23(long exposure)
Med23(low exposure)
Actin(long exposure)
Actin(low exposure)
GAPDH(long exposure)
GAPDH(low exposure)

Fig.5E

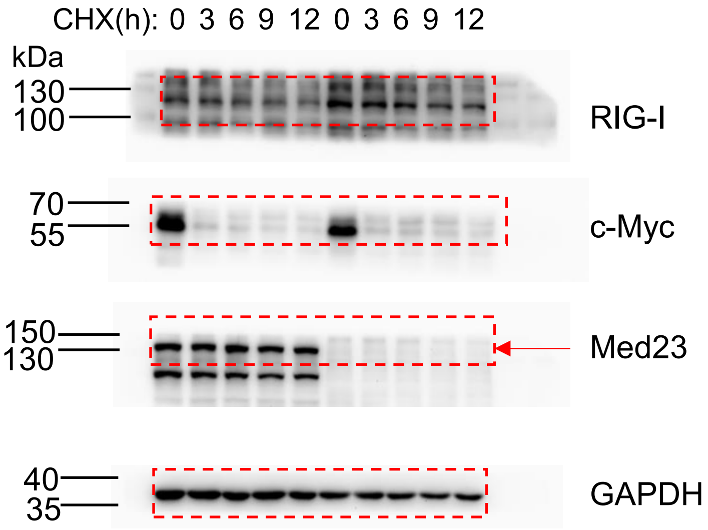
CHX(h): 0 3 6 9 12 0 3 6 9 12
kDa
130
100
RIG-I
70
55
c-Myc
150
130
Med23
40
35
GAPDH

Fig.S4A

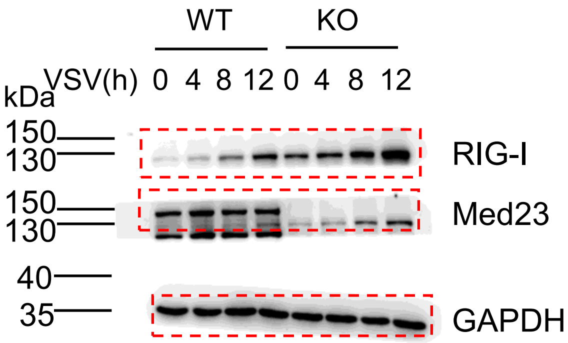
WT
KO
VSV(h) 0 4 8 12 0 4 8 12
kDa
150
130
RIG-I
150
130
Med23
40
35
GAPDH

Fig.S4B

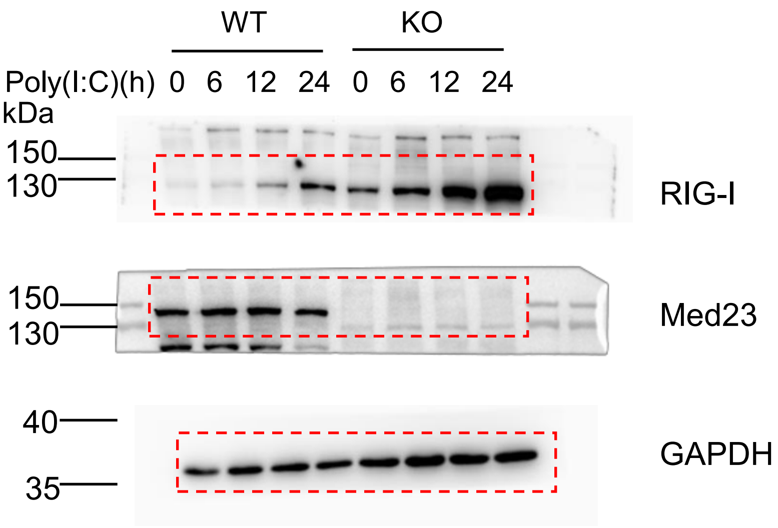

WT
KO
Poly(I:C)(h) 0 6 12 24 0 6 12 24
kDa
150
130
RIG-I
150
130
Med23
40
35
GAPDH

Fig.S4C

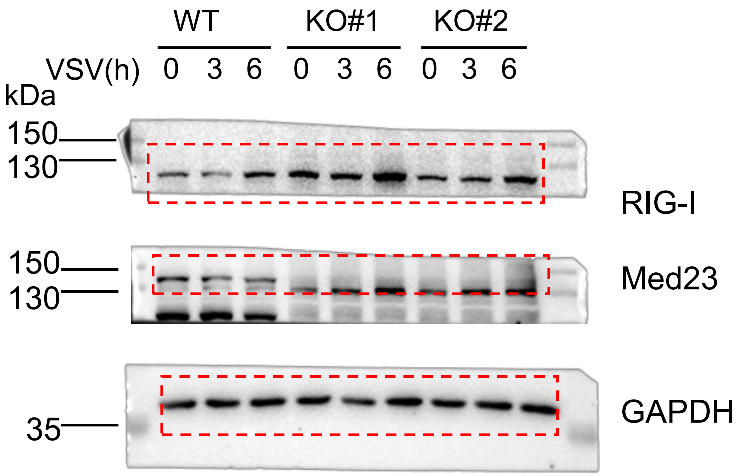
WT
KO#1
KO#2
VSV(h)
0 3 6 0 3 6 0 3 6
kDa
150
130
RIG-I
150
130
Med23
35
GAPDH

Fig.S4D

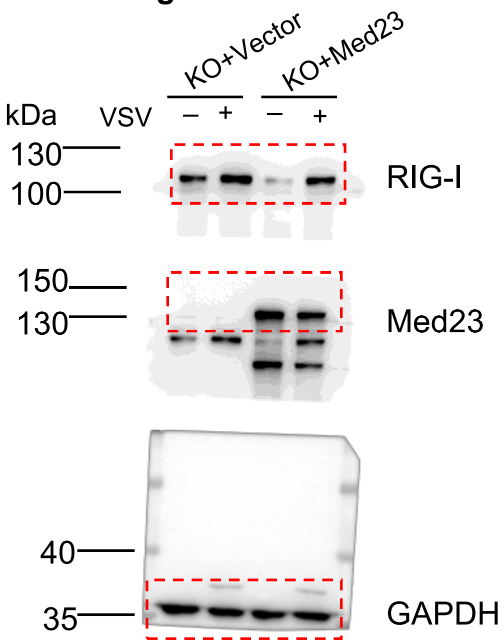
KO+Vector
KO+Med23
kDa
VSV
–
+
–
+
130
100
RIG-I
150
130
Med23
40
35
GAPDH

Fig.6A

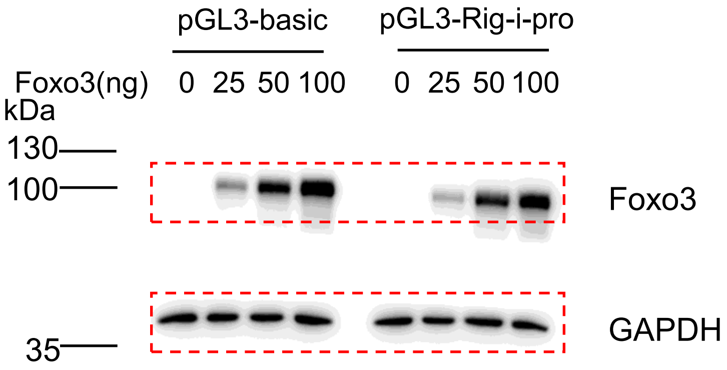
pGL3-basic
pGL3-Rig-i-pro
Foxo3(ng) 0 25 50 100 0 25 50 100
kDa
130
100
35
Foxo3
GAPDH

Fig.6B

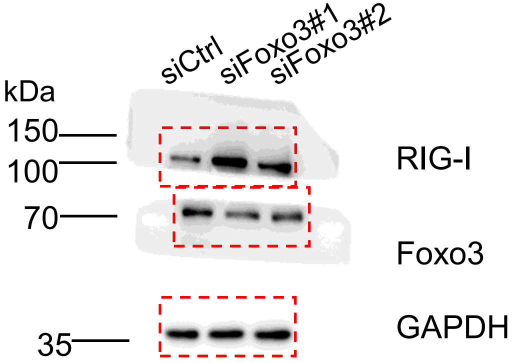

Fig.6D

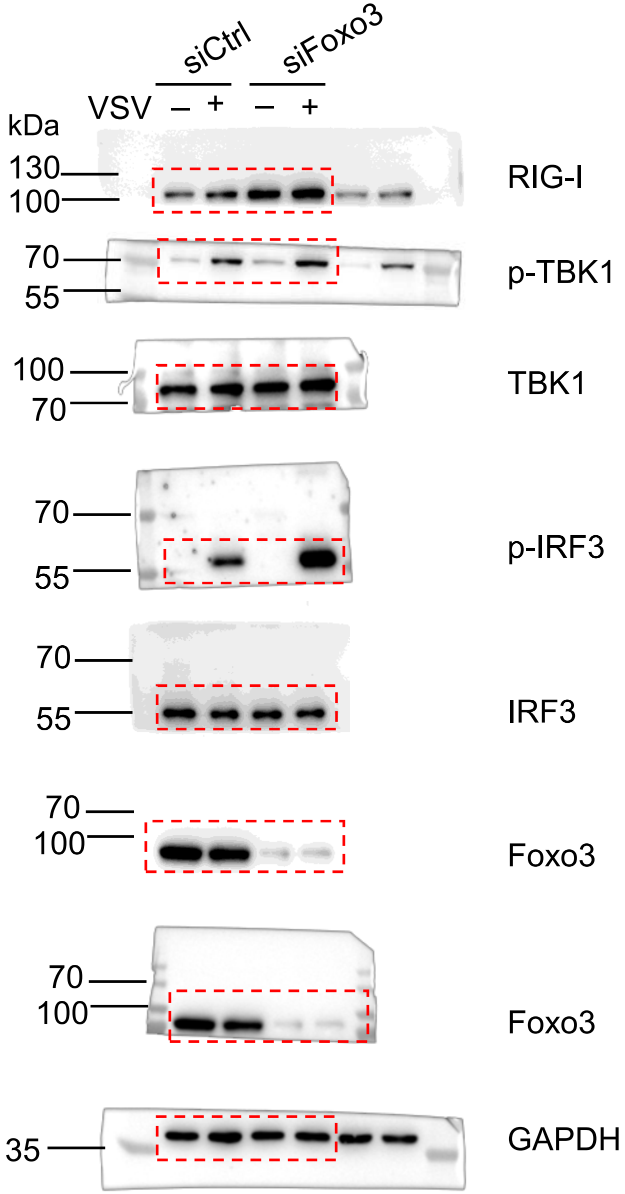
siCtrl
siFoxo3
VSV – + – +
kDa
130
100
RIG-I
70
55
p-TBK1
100
70
TBK1
70
55
p-IRF3
70
55
IRF3
70
100
Foxo3
70
100
Foxo3
35
GAPDH

Fig.6F

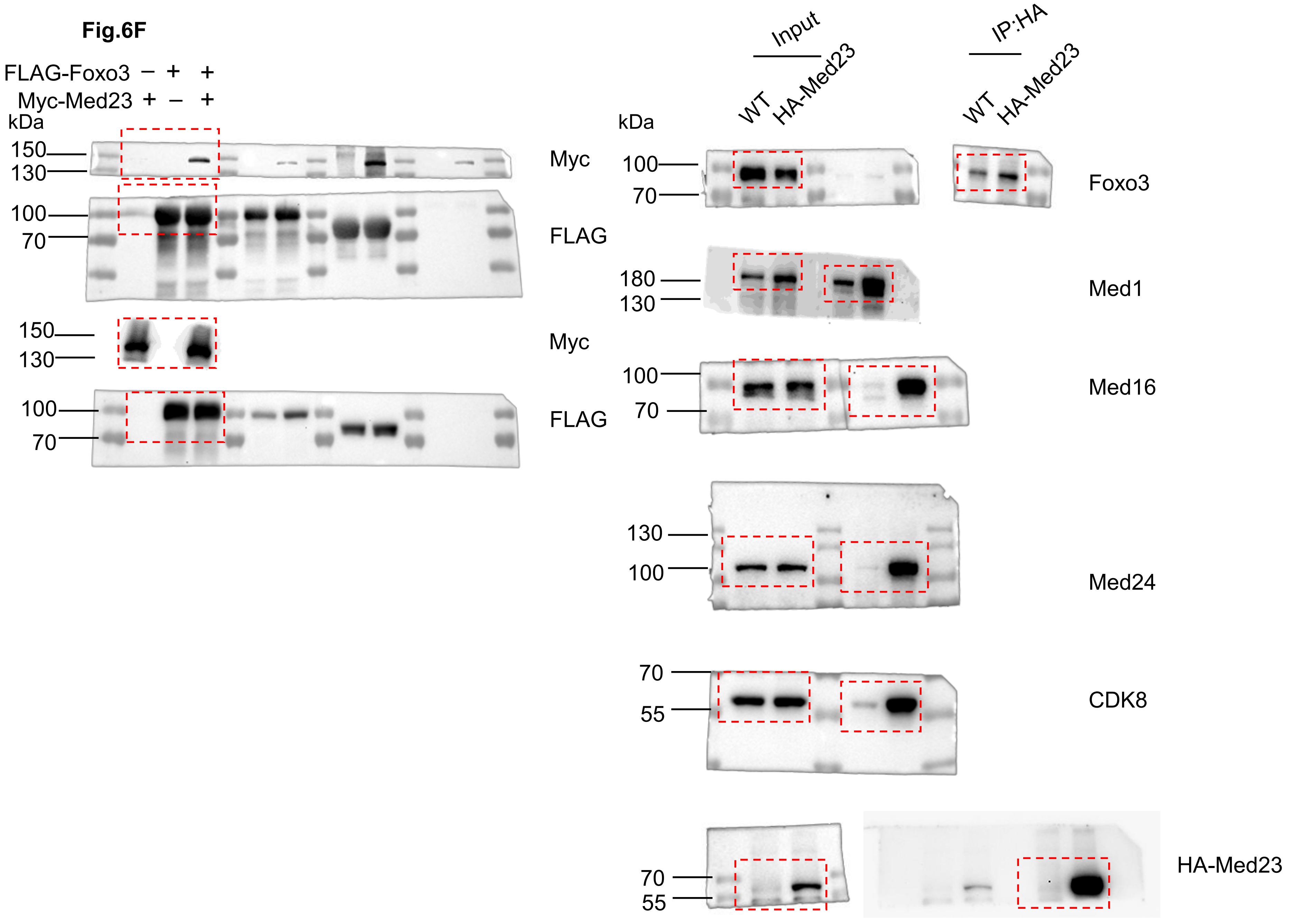
FLAG-Foxo3 – + +
Myc-Med23 + – +
kDa
150
130
100
70
150
130
100
70
Myc
FLAG
Myc
FLAG
Input
IP:HA
WT
HA-Med23
WT
HA-Med23
kDa
100
70
180
130
100
70
130
100
70
55
70
55
Foxo3
Med1
Med16
Med24
CDK8
HA-Med23

Fig.S5C

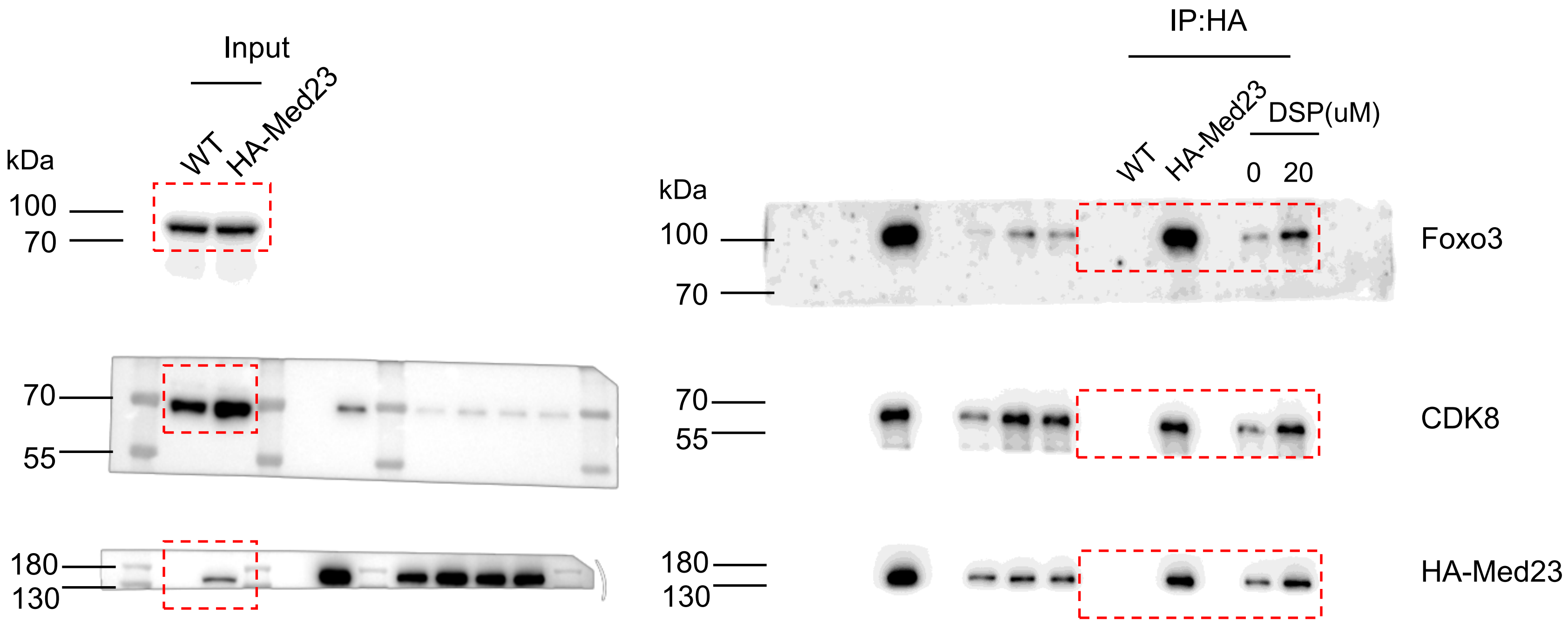
Input
WT
HA-Med23
kDa
100
70
70
55
180
130
IP:HA
WT
HA-Med23
DSP(uM)
0
20
kDa
100
70
Foxo3
70
55
CDK8
180
130
HA-Med23
