# Supplementary material for: Mediator complex subunit MED23 dampens antiviral innate immunity by restricting RIG-I expression
Source: PLoS Biol. 2025 Jul 24;23(7):e3003294. doi: 10.1371/journal.pbio.3003294 (PMC12316392; doi:10.1371/journal.pbio.3003294)
Supplement: Attachment — Submitted filename: Response to Reviewers.pdf [file pbio.3003294.s015.pdf]

Reviewer #1:

Using a combination of biochemical and molecular analyses as well as ex vivo cultured mammalian cells and tissue-specific MED23 knockout mouse models, Xiong et al. provide important insights into the role of Med23 and FOXO3 in regulating the innate immune response via RIG-I in mammals. The manuscript is well-written, with a logical experimental design and solid data. The findings are novel and improve our understanding of the molecular mechanisms underlying innate immunity. Below are a few points that could further strengthen the study.

We sincerely appreciate the positive assessment of our work and constructive suggestions. Below are our point-to-point responses.

Major Points:

First, it is unclear why MED23 deletion reduces FOXO3 binding to the RIG-I promoter (Fig. 6G). Does the loss of MED23 affect FOXO3 mRNA or protein levels? Additional evidence or discussion to clarify this mechanism would help the understanding of this observation.

We sincerely appreciate your insightful question regarding the potential regulatory role of Med23 in Foxo3 expression. To address this, we first assessed Foxo3 mRNA and protein levels under basal and VSV-stimulated conditions. Med23 deletion does not alter the expression levels of Foxo3 mRNA or protein (Figure R1. A-B). In uninfected BMDMs, $Med23^{\Delta M}$ had no significant effect on Foxo3 mRNA or total protein levels compared to $Med23^{f/f}$. Upon VSV infection, Foxo3 mRNA and protein expression decreased, consistent with prior reports of stress-induced Foxo3 degradation (Litvak et al. 2012). Importantly, Foxo3 levels in $Med23^{\Delta M}$ BMDMs remained comparable to $Med23^{f/f}$ BMDMs, suggesting that MED23 does not regulate the FOXO3 expression.

Regarding why Med23 deletion reduces Foxo3 binding to the RIG-I promoter,

we thought that Mediator Med23, as a transcriptional cofactor, may enhance the cooperative binding of Foxo3 to RIG-I promoter; whereas Med23 deficiency may compromise the Foxo3 recruitment to the RIG-I promoter. A similar example is a recent study showing that FOXA1 binding is strongly affected by co-transcription factor AP-1 and CEBPB (Xu et al. 2024). This possibility has now been included in the revised Discussion section.

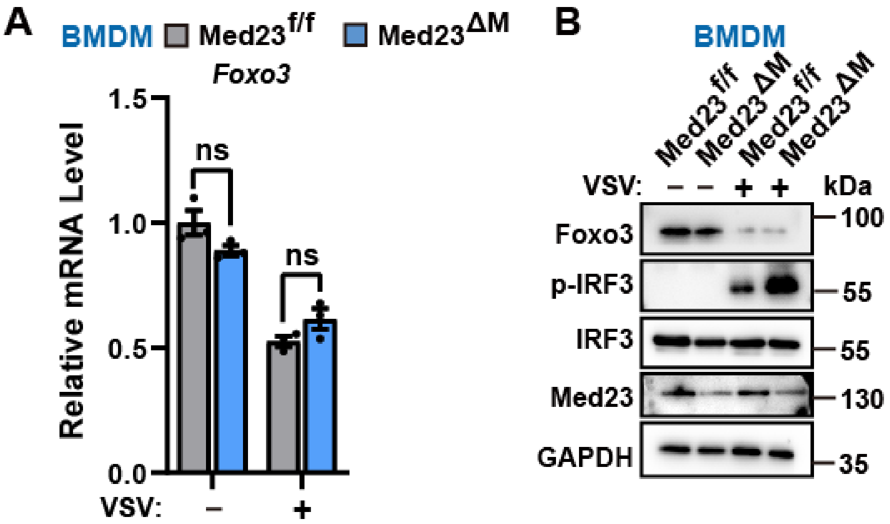

Figure R1. (A) qPCR analysis of the mRNA levels of Foxo3 in Med23$^{f/f}$ and Med23$^{\Delta M}$ BMDMs (n=3) infected with VSV (MOI=0.5, n=3) for 4h. Expression levels were normalized to GAPDH mRNA and then to the Med23$^{f/f}$ BMDMs without VSV infection. (B) Immunoblot analysis of the indicated proteins in Med23$^{f/f}$ and Med23$^{\Delta M}$ BMDMs infected with VSV (MOI=0.5) for 4h. Statistical analysis in A was performed by two-tailed unpaired Student's t test; ns, not significant.

Second, the interaction between Med23 and FOXO3 is supported by co-IP experiments. However, co-IP does not exclude the possibility of indirect interactions. Given the importance of this point, additional experiments, such as a GST pulldown assay or alternative methods, could provide more direct evidence for the physical interaction between these two proteins.

To validate the direct interaction between Med23 and Foxo3, we performed DSP crosslinking-coupled co-IP experiments to discriminate the direct from the indirect interactions. As shown in Figure R2 (or Fig.S5C), the interaction between Med23 and Foxo3 was retained following DSP crosslinking and high-

stringency washes, thus providing robust evidence of direct binding. This finding aligns with our co-IP data (Fig.6F), which collectively demonstrate the Med23-Foxo3 link in regulating the innate immune response.

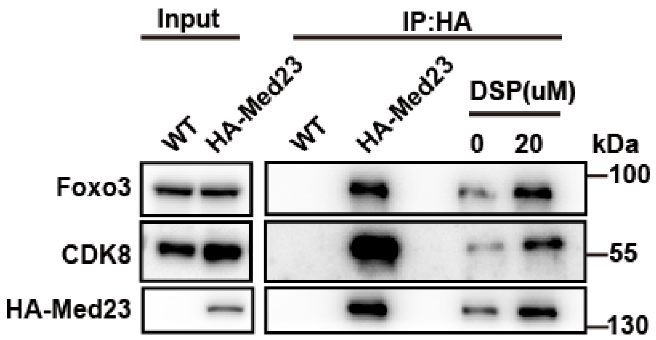

Figure R2. Immunoblot analysis of endogenous Foxo3 in the cell lysates of HA-Med23-KI HEK293T cells treated with DSP (20uM) immunoprecipitated with the HA antibody.

Minor Points:

First, the rescue experiments involving ectopic Med23 expression in Med23 KO cells are critical. However, it is unclear whether the ectopically expressed Med23 is comparable in levels to endogenous Med23. This applies to Fig. 1C and Fig. S3H. Clarifying this point would strengthen the conclusions.

Our Western blot data (Figure R3) confirm that ectopic Med23 expression in the stable rescue cell lines exceeds the endogenous levels, a recognized technical limitation in achieving precise expression control in overexpression systems. Notably, we previously found that the overexpressed Med23 could either incorporate into the Mediator complex insufficiently or competitively inhibit the overall function of the Mediator complex, a sequestering effect (Liu et al. 2016, Wang et al. 2019), which is consistent with the partial phenotypic rescue effect observed here. Importantly, expression titration experiments with Med23 confirm the dosage-dependent rescue phenotypes (Fig.S1I), thus supporting the Med23-specific effect.

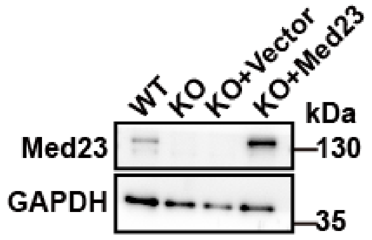

Figure R3. Immunoblotting to detect Med23 in WT, Med23 KO, and Med23 knockout MEFs reconstituted with Vector or Med23 overexpression.

Second, the authors conclude that "Med23 deficiency enhances the innate immune response for viral clearance and minimizes tissue damage in vivo" (Fig. 3; page 11). This conclusion might be an overinterpretation, as the observed effects could also result from Med23's effect on viral replication. Additional evidence to distinguish between these possibilities or revised wording would be appropriate.

Thanks for highlighting this important point. We agree that the observed effects potentially be attributed to Med23's effect on viral replication, in addition to its role in modulating the innate immune response. In response to Reviewer #2’s suggestion, we investigated whether Med23-deficient mice are protected from VSV-induced diseases, such as weight loss and mortality. As now shown in Figure 3F-G, Med23$^{\Delta M}$ mice displayed significantly reduced weight loss and improved survival following VSV infection compared to Med23$^{f/f}$ mice. These findings support the notion that Med23-deficient mice were protected against viral pathogenesis, although a potential contribution from reduced viral replication cannot be excluded. Accordingly, we have now revised the conclusion to more accurately reflect the data: "Collectively, these results suggest that Med23-deficient mice showed an enhanced innate immune response to RNA virus infection, limited viral replication and increased survival."

Third, on page 12, the authors stated, "These downregulated genes in Med23$^{\Delta M}$ BMDMs were enriched in the cell cycle and DNA replication, but

Med23 deficiency did not affect the population of BMDMs (data not shown)." It is unclear what analyses were conducted to conclude that Med23 mutation has no effect on BMDM populations. Did the authors assess cell cycle profiles to determine the absence of effects on cell cycle progression? If so, further explanation of how these findings align with the observed gene expression changes would be valuable. Adding these data in the Supplementary data would be helpful.

To clarify how we concluded that Med23 deficiency does not affect BMDM populations, we have the data in Figure R4 or revised Fig. S2C. We performed the flow cytometry analysis of BMDMs from Med23$^{f/f}$ and Med23$^{\Delta M}$ mice, which revealed no significant difference in the proportion of F4/80^{+}CD11b^{+} cells. This confirms that Med23 deficiency does not alter the overall BMDM population under steady-state conditions (Figure R4). While RNA-seq data showed downregulation of cell cycle/DNA replication-related genes, the absence of phenotypic changes in BMDM abundance suggests that these transcriptional changes may be "buffered" at the protein level, thus preventing phenotypic effects.

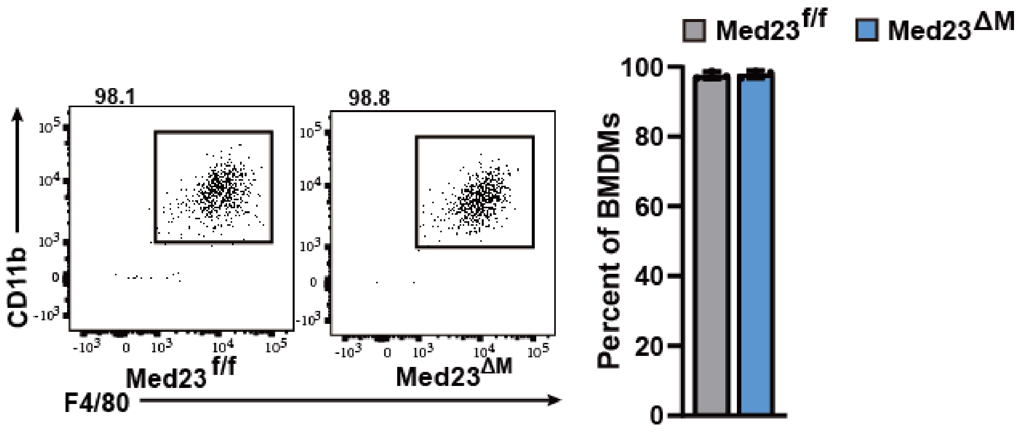

Figure R4. Flow cytometric analysis of the percentages of BMDMs from Med23$^{f/f}$ and Med23$^{\Delta M}$ mice (n=3).

Fourth, the authors noted that "numerous Foxo3 target genes were upregulated in Med23 knockout cells" (page 18). Including a Venn diagram to

show the overlap of these genes with FOXO3 targets and their relative proportions would provide a clearer representation of these findings.

As seen in Figure R5 or revised Fig.S5D, we have included a Venn diagram to visualize the 52 genes in Fig.6H, which show the overlap gene set between those upregulated in Foxo3 KO cells under poly(I:C)-treated or untreated conditions (Litvak, Ratushny et al. 2012), and those induced by VSV infection in our RNA-seq analysis in this study.

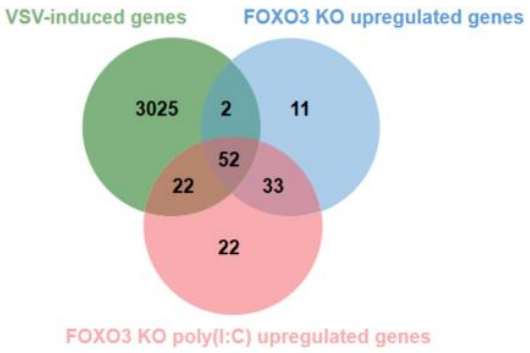

Figure R5. Venn diagram to show the overlap of these genes upregulated in Foxo3 KO cells treated with or without poly(I:C) and those induced by VSV infection in our RNA-seq analysis.

Finally, in the Discussion section, the authors could elaborate on the potential role of other Mediator subunits in the described processes. For instance, in Med23 KO cells, does FOXO3 still require interaction with the remaining subunits of the Mediator complex to activate the transcription of RIG-1 gene? Additionally, how does the Mediator complex (with Med23) shift from repressing to activating RIG-1 transcription? The authors could also discuss whether Med23 binding to FOXO3 or the rest of the Mediator complex is regulated by viral infection. Furthermore, it would be helpful to discuss whether the model presented in Fig. 7 also explains the effects of Med23 loss on TBK1 and IRF3 phosphorylation, as well as IRF3 nuclear translocation, as shown in Fig. 1.

Thank you for these insightful questions, which have prompted us to deepen the mechanistic discussion. The revised manuscript has now includes the following consideration or changes.

a) While our current study revealed that Med23 interacts with the transcription factor Foxo3, we cannot rule out the possibility that Foxo3 may interact, directly or indirectly, with other Mediator subunits in Med23 KO cells. However, other possibilities include that additional transcription factor(s)/cofactor(s) may contribute to activate the transcription of RIG-I gene in the absence of Med23.

b) Although Mediator is well recognized as a transcriptional coactivator, several studies suggest that several Mediator subunits, especially the kinase submodule, can also inhibit gene transcription (Akoulitchev et al. 2000, Ding et al. 2008). For example, Med19 and Med26 link the RE1 silencing transcription factor (REST) with the G9a histone methyltransferase, which suppresses neuronal gene expression through histone H3K9 methylation in nonneuronal cells (Ding et al. 2009). In this study, we observed that Med23 suppresses RIG-I through Foxo3-mediated transcriptional repression. In a previous study, Foxo3 represses gene expression by forming a trimeric complex with nuclear corepressor 2 (NCOR2) and histone deacetylase 3 (HDAC3), leading to a reduction in histone H4 acetylation (H4AC) on target gene promoters (Litvak, Ratushny et al. 2012). There is a possibility that Foxo3 might repress RIG-I expression by collaborating with Mediator Med23 as well as other repressors/corepressors. When Med23 is depleted, the repressed RIG-I is relieved and becomes activated. In the future, it will be interesting to define the dynamic regulated network within which Mediator Med23 coordinates the antiviral signaling and to determine exact molecular mechanisms underlying the antiviral immune response.

c) Regarding whether Med23 binding to FOXO3 or the rest of the Mediator complex is regulated by viral infection, as shown in Figure R1, Foxo3 mRNA or protein levels decrease in BMDMs following VSV infection. Based on this, we thought that the interaction between Med23 and Foxo3 may also be downregulated by viral infection. Consistent with this idea, the

interaction between endogenous HA-Med23 with Foxo3 in 293T cells was reduced upon VSV infection (Figure R6). Noticeably, the Foxo3 levels did not decrease in the input samples under VSV infection in 293T. This discrepancy between BMDMs and 293T may be due to differences in cell type.

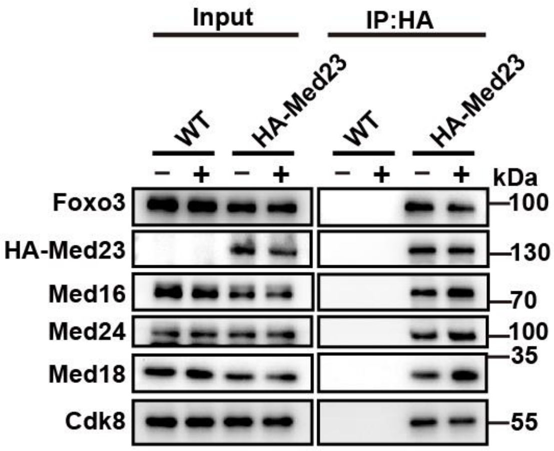

Figure R6. immunoblot analysis of endogenous Foxo3 and Mediator subunits in the cell lysates of HA-Med23-KI HEK293T cells immunoprecipitated with the HA antibody.

d) In response to the reviewer's suggestion, we have updated the model (Figure R7 or revised Fig.7) to incorporate the effects of Med23 deficiency on TBK1 and IRF3 phosphorylation.

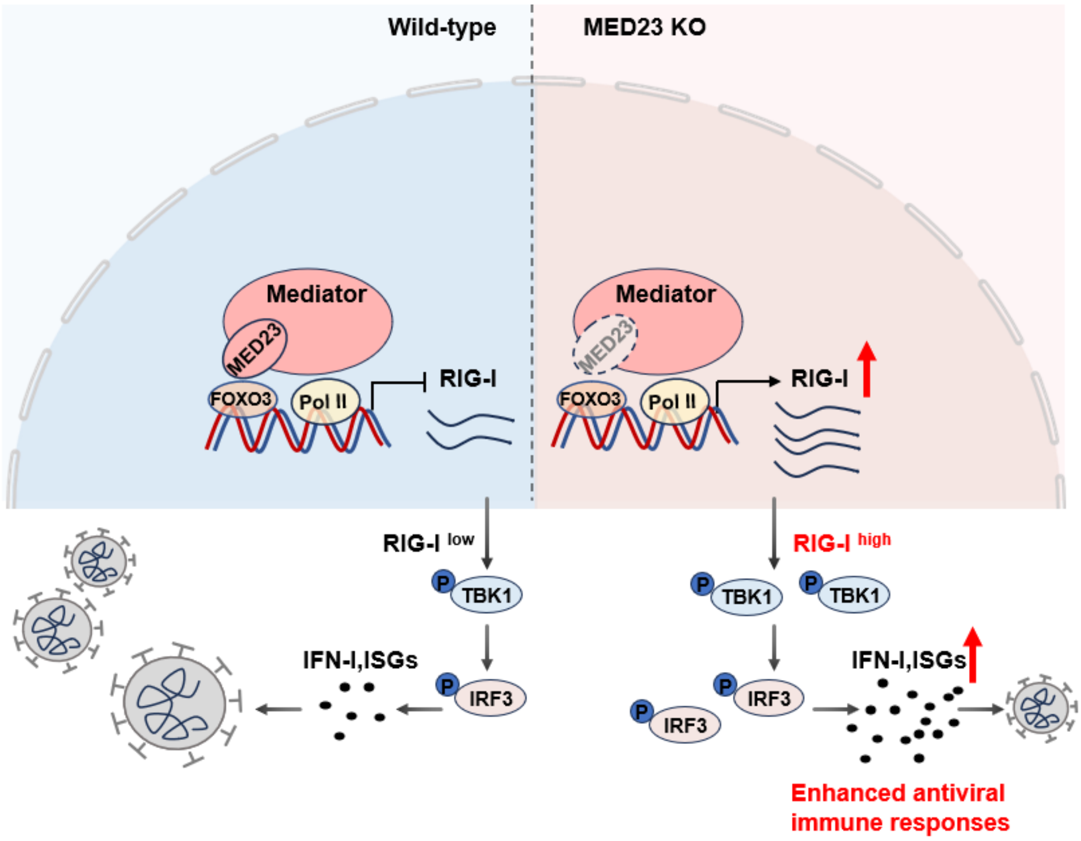

Figure R7. Schematic showing that Med23 inhibits the antiviral IFN-I response by negatively regulating RIG-I. Med23 interacts with Foxo3 to transcriptionally repress RIG-I, thereby inhibiting the RNA virus-induced phosphorylation of TBK1 and IRF3, suppressing the antiviral innate immune response, and promoting virus replication.

Reviewer #2:

The study focuses on an intriguing aspect of innate immune regulation during RNA virus infection, identifying Med23 as a key regulator of the RIG-I-dependent antiviral response. The authors utilize a range of methodologies, multiple cell lines, and a transgenic mouse model to provide insights into this critical regulatory mechanism. However, the manuscript is hindered by several issues, including insufficient details on experimental rationale and design, statistical analyses, inconsistent labeling, and mislabeled panels. These issues make it significantly difficult to evaluate the study's

quality and scientific rigor.

We sincerely appreciate the reviewer's constructive comments. We have further clarified the experimental rationale and design, statistical analyses, and have thoroughly rechecked all figures, panels, and text. Below are our point-by-point responses.

Major Points:

1. Critical experimental designs and conditions, such as VSV infection and poly(I:C) treatment, are not adequately described. These methods are fundamental to stimulating RIG-I and must be clearly stated for scientific rigor. Additionally, statistical analysis needs to be explicitly described in the figure legends, along with the number of experimental repeats. The lack of this information compromises the scientific integrity and hinders a thorough review of the manuscript. Here are some examples,

Fig. 1C: Reconstitution methodology (e.g., transfection) is not described.
Experimental conditions (MOIs, treatment times) are absent for Figs. 1D-E.
Fig. 5 lacks information on siRNA procedures, CHX concentration, and other experimental details.
Fig. 6A: Foxo3 transfection details (e.g., plasmid amount, time point) and the units in the panel are missing.
Fig. 6F: The rationale behind analyzing additional proteins (Med1, Med16, etc.) is not explained in the text or figure legend.
Fig. 6H: Experimental design details are absent, making interpretation difficult.
No description of the y-axis label "relative mRNA level" in qRT-PCR panels.

We fully agree that transparent reporting of experimental details is essential. Below, we address each concern and confirm that all requested information has been added to the revised manuscript:

a) VSV MOI and poly(I:C) concentration are now explicitly stated in the figure legends. All figure legends now clarify the statistical tests and the

biological replicates performed.

b) In Fig. 1C (revised Fig.1E) legend: the reconstitution methodology is added: " Immunoblotting to detect Med23 in Med23 knockout MEFs reconstituted with pMSCV-Med23 retroviral overexpression system." Experimental conditions (MOIs, treatment times) are now explicitly stated in the figure legends of Figs. 1D-E (revised Fig.1F-G).

c) In the figure legend of Fig. 5C-E: siRNA concentration 50 nM (Fig.5C-D) and CHX treatment 50 ng/mL (Fig.5E) was clarified.

d) Fig. 6A: transfection time point (48 hr post-transfection) and plasmid amount are specified in the legend, and plasmid units (ng) are added to the axis label.

e) Fig. 6F: Explained in the text: "Importantly, endogenous HA-Med23 interacts not only with Mediator complex as indicated by Med1, Med16, Med24 and CDK8 but also associates with Foxo3."

f) Fig. 6H: We have included a Venn diagram to visualize the overlap between Med23 KO-upregulated genes and known FOXO3 target genes (Fig.S5D).The 52 genes in Fig.6H represent the intersection of genes upregulated in FOXO3 KO cells treated with or without poly(I:C) (Litvak, Ratushny et al. 2012) and those induced by VSV infection in our RNA-seq analysis. Heatmap analysis revealed the expression profiles of the 52 genes in the RNA-Seq data from Med23 knockout MEFs.

g) the "relative mRNA level" in the qRT-PCR panels refers to transcript levels normalized to GAPDH, as described in the RNA isolation and quantitative reverse transcription PCR (qRT-PCR) section of Materials and Methods.

2. It is unclear whether stable Med23 KO cells, such as MEFs, exhibit baseline levels of certain IFN-I or cytokines. For instance, in Figs. 1A and 1B, Il6 shows a higher level but no significant increase in response to VSV and poly(I:C) treatments. Clarification is needed.

In Fig. 1A and 1B, we indeed observed elevated basal Il6 mRNA levels in

Med23 KO MEFs compared to WT under unstimulated conditions. We have also added significance markers for the differential basal IFN-I mRNA levels. However, the basal levels of IFN-I or cytokines in both WT and Med23 KO cells are significantly lower compared to the VSV-stimulated levels. Thus, we did not focus on investigating the basal level differences.

3. Although qRT-PCR is used to assess viral load in Figs. 1, 2F-G, and 3D, as well as cytokine mRNA levels, plaque assays, and ELISA, are more reliable and should be included to confirm viral load quantification and released cytokines.

Thank you for your suggestion. We have performed ELISA assays to quantify IFNβ protein levels (revised Fig.1C-D and Fig.S1B-C) and carried out the $TCID_{50}$ analysis to measure infectious viral titers (revised Fig.S1N-O, Fig.2F-G and Fig.3D). These results align well with our qRT-PCR data.

4. Figs. 1H and 1J: A strong increase in nuclear IRF3 after VSV infection is observed. However, this change is inconsistent with Fig. 1H. The rationale for this discrepancy needs to be addressed.

The results in Fig. 1H (now revised Fig. 1J) were derived from MEFs, while those in Fig. 1J (now revised Fig. 1L) used HeLa cells. The apparent discrepancy between these figures arises from potential cell-type-specific regulatory mechanisms. However, we should point out there were consistently increased p-IRF3 in KO cells upon VSV or poly(I:C) treatment.

5. In Fig. S1J, Med23 is induced by poly(I:C), but this observation is inconsistent with Fig. 1H. This contradiction requires clarification.

This may result from uneven protein loading, as indicated by the higher LaminB1 levels in poly(I:C)-treated sample compared to untreated control (Fig. S1J and Fig. 1H, now revised Fig. S1M and Fig1J). Overall, Med23 protein levels are unchanged upon either poly(I:C) or VSV stimulation, as demonstrated in multiple experiments in Fig.1J-K, S1K-L,2D-E,5B and S4A-D.

6. Fig. 2A: The presence of three lanes per mouse is unexplained. Additionally, KO cells exhibit basal Med23 protein expression. Is this due to incomplete knockout or contamination by non-myeloid cells? This requires explanation.

In Fig. 2A, each set of three lanes represents biological replicates from three individual littermate mice. The faint Med23 signal observed in KO cells may derive from non-myeloid cells, such as osteoclasts (Mun et al. 2020, Ren et al. 2023).

7. Fig. 1A shows strong increases in IFN-I and cytokine levels in MEF KO cells compared to WT, whereas Figs. 2B-C show moderate increases in BMDM and PEM. If infection conditions are comparable, why are the effects less pronounced in BMDM and PEM? Missing VSV infection details impede proper evaluation.

First, differences in IFN-I and cytokine induction between MEFs and BMDM or PEM may result from (1) distinctive cell lines, as MEFs are immortalized embryonic fibroblasts, whereas BMDM/PEM are primary macrophages derived directly from mice; (2) different infection conditions: MEFs were infected at MOI = 1, while BMDMs or PEMs were infected at MOI = 0.5 (now clarified in the figure legends). Despite of the different magnitude of response, Med23 deletion consistently enhanced antiviral responses across different cell types, supporting its specific regulatory role in innate immunity.

8. Fig. 2E: VSV infection conditions and infection times are not described. Furthermore, why does VSV infection reduce IRF3 levels in this context? The basal expression of Med23 in KO cells also needs to be addressed.

As suggested, we have added VSV infection conditions and infection times (MOI = 0.5, 4h) to the Fig. 2E legend. The observed IRF3 reduction post-infection may reflect virus-triggered IRF3 degradation (Liu et al. 2024). Residual Med23 in PEMs from Med23$^{\Delta M}$ mice may arise from non-myeloid cells, such as B cells or T cells as indicated in the literature (Ray and Dittel

2010).

9. Fig. 3: Given a stronger innate response, does Med23 KO protect mice from VSV-induced diseases, such as weight loss or survival deficits?

Per suggestion, we have monitored body weight in Med23$^{f/f}$ and Med23$^{\Delta M}$ mice following intraperitoneal injection with a low-dose VSV, and survival after high-dose VSV infection. As now shown in Figure R8A-B, Med23$^{\Delta M}$ mice exhibited significantly reduced weight loss and improved survival post-VSV infection compared to Med23$^{f/f}$ mice, further supporting that enhanced innate immunity in conditional Med23 knockout mice confers protection against viral pathogenesis. We have now included these results in the revised manuscript as Fig.3F-G.

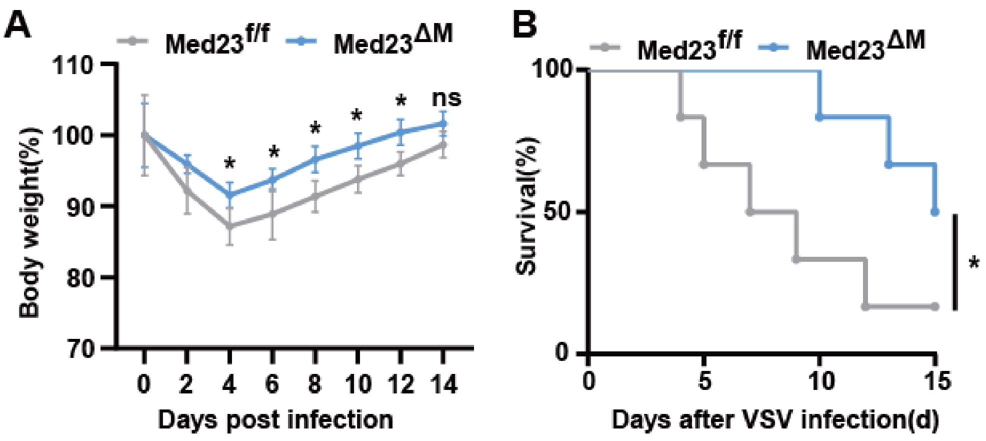

Figure R8. (A) body weight was monitored in Med23$^{f/f}$ and Med23$^{\Delta M}$ mice injected intraperitoneally with VSV (2×10^7 PFU per mouse, n=4) for 14 days. (B) Survival (Kaplan-Meier curves) was monitored in Med23$^{f/f}$ and Med23$^{\Delta M}$ mice injected intraperitoneally with VSV (1×10^8 PFU per mouse, n=6) for 15 days. Statistical analysis in (A-B) was performed by two-tailed unpaired Student's t test; ns, not significant, $^*P<0.05$.

10. Figs. S3F-G: If the KO cells exhibit higher basal ISRE activity, the statistical significance should be shown, and its implications discussed.

We have added statistical significance labels to the basal ISRE activity in KO cells (revised Fig. S3G-H). Elevated basal ISRE activity suggests elevated

innate immune signaling in MED23 KO HeLa cells, which might be triggered by the steady state factors in the culture media.

11. Fig. 5I and 6G: Limited information about the ChIP experiments is provided. There is no description of why these sites were chosen for analysis and the location of the promoter. More importantly, why are the sites analyzed different from these two experiments?

In Fig. 5I, the analyzed sites were selected based on Pol II occupancy peaks identified in public ChIP-seq datasets (ENCODE). For Fig. 6G, primers were designed to target predicted FOXO3-binding motifs (via JASPAR) within the RIG-I promoter. The description has been added to the Materials and methods section of the manuscript.

12. Fig. 5 and Fig. S4: Immunoblotting results show consistently higher basal RIG-I protein levels in Med23 KO cells across experiments, yet qRT-PCR does not reflect increased mRNA levels. This suggests that there are additional regulations that cause higher basal protein levels. Do the authors have any comments on the higher protein levels?

Our data demonstrate that RIG-I protein levels are elevated in uninfected Med23 KO cells across multiple cell types (BMDMs, MEFs, and RAW264.7 cells), as shown in Figures 5B, S4A-C. RIG-I mRNA levels tended to increase in MEFs (Fig. S4F-G) and BMDMs (Fig. 4A and 5F) in uninfected cells. We thought that this discrepancy may reflect the posttranscriptional regulation of basal level of RIG-I. Also, there might be unknown factor(s) in the steady state condition triggering the differential basal expression.

In order to understand the elevated basal RIG-I protein levels in Med23 KO cells, we first treated WT and Med23 KO cells with cycloheximide (CHX), a protein biosynthesis inhibitor, which revealed comparable RIG-I protein degradation rates between WT and Med23 KO cells (Fig. 5E). In addition, as reviewer #4 suggested, actinomycin D (Act D) chase assays were carried out

and confirmed unchanged RIG-I mRNA stability in Med23 KO cells (revised Fig.4E or Figure R9). These results reinforce that Med23 regulates RIG-I mainly at the transcription level, as demonstrated by enhanced RIG-I promoter luciferase activity (Fig. 5G) and increased Pol II recruitment to the RIG-I promoter (Fig. 5H) in KO cells. Overall, we agree with the reviewer that there might be additional regulations that cause higher basal protein levels. Since our current study focuses on how Med23 regulates the stimulated innate immunity, future studies may further dissect the underlying mechanisms of the differential basal levels.

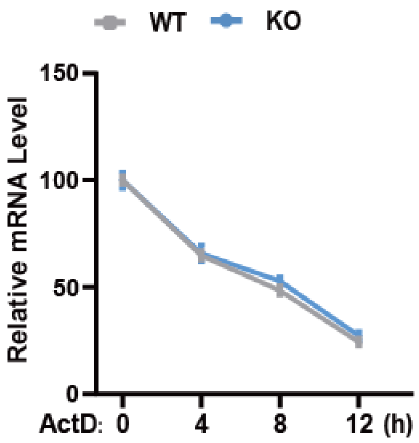

Figure R9. qPCR analysis of RIG-I in WT and Med23 knockout HeLa cells (n=3) treated with Act D (1mM/mL) for 0,4,8,12h.

Minor Points:

1. Fig. 1 lack WB for Med23 KO mice.

We sincerely apologize for the carelessness. We confirm that the Med23 Western blot data have now been added to Fig. S1A.

2. Fig. S1: Clarify whether the KO cell lines are single clonal.

The Med23 KO cell lines used in Fig. S1 are derived from single-cell clones. And we clarified that Med23 knockout HeLa and RAW264.7 cell lines were generated using the CRISPR/Cas9 plasmid vector PX330, and single-cell clones were subsequently isolated by FACS in the Materials and methods.

3. Use consistent terminology: Type I IFN or IFN-I.

We have revised the manuscript to consistently use “IFN-I”.

4. Protein markers for molecular weights are missing in immunoblots.

Protein molecular weight markers have been added to all immunoblots in the revised figures.

5. In Fig. 1C, "Gapdh" should be capitalized.

We have corrected "Gapdh" to "GAPDH" in all immunoblotting results.

6. The statement in Line 219 regarding elevated IFN-β and Il6 mRNA levels in Med23$^{\Delta M}$ mice liver is inconsistent with Fig. 3C, where no statistical significance is shown.

We have revised the statement in the text to clarify that: "Consistent with the in vitro findings, qPCR analysis revealed elevated levels of IFNβ and IL6 mRNA in the spleen and lungs of Med23$^{\Delta M}$ mice than in those of Med23$^{f/f}$ mice, with a similar trend observed in the liver. "

7. Rsad2 is not a cytokine (Line 262).

We have corrected the text.

8. Fig. 4: Gene ontology analysis should be included to provide insights into the role of the downregulated genes in Med23 KO cells, with or without VSV infection.

We have performed Gene Ontology analysis on the downregulated genes in Med23$^{\Delta M}$ BMDMs with and without VSV infection and included these data in Fig.S3B. The most highly enriched downregulated pathways in BMDMs from Med23$^{\Delta M}$ mice were associated with cell cycle and DNA replication, including “regulation of cell cycle process”, “activation of the pre-replicative complex” and “DNA replication”.

9. Fig. 4E: Data presented as a heatmap would benefit from statistical

analysis and fold change representation for greater interpretability.

To enhance clarity, we have revised the Fig.4E legend to: “Heatmap showing the relative expression values (Z score) of differentially expressed ISGs according to the RNA-seq data, ranked by fold change”.

10. Fig. 6G is mislabeled as Fig. 6I in the figure legend.

This has been corrected in the figure legend.

11. Line 280: The statement "Med23 interferes with innate immune signaling by limiting ISG expression during VSV infection" should be revised for clarity to: "Med23 interferes with innate immune signaling, resulting in limited ISG expression during VSV infection."

As suggested, we have corrected the statement in the revised manuscript.

Reviewer #3:

The manuscript by Xiong et al. reports the transcriptional level regulation of RNA virus sensor RIG-I by Mediator complex subunit 23 (Med23). Using different cell lines and primary cells from mice, they provided convincing evidence that Med23 negatively regulates type I interferon, inflammatory cytokines and chemokines in the context RNA virus infection or mimicry. In vivo, the authors provided a physiological relevance that Med23 represses type I interferon thus dampening anti-viral response. Overall, they presented a model that Med23 collaborates with Foxo3 to suppress RIG-I leading to downregulated type I interferon response.
Although transcriptional level regulation of innate immune sensors, inflammatory cytokines and chemokines by mediator seems novel, they author oversimplified the model by drawing a single direction arrow from RIG-I to type-I interferon and other ISGs. However, since RIG-I (DDX58) is also an ISG,

the reduced level of type I interferon is expected to lead to lower RIG-I level. Biased by their model, the authors did not address the possibility that Med23 directly regulate type I interferon or other inflammatory cytokines. Indeed, in the absence of viral infection, Med23$^{\Delta M}$ already showed an increase of various ISGs (Figure 4B and E), indicating the regulation of ISGs might be independent of RIG-I's recognition of viral RNA. Given the broad role of mediator complex, the major scientific question of this manuscript will be better addressed with unbiased ChIP-seq approach.

We sincerely thank you for raising these critical points. To address concerns about simplification and potential indirect effects, we have revised the model in Figure 7 to integrate the effects of Med23 loss on TBK1 and IRF3 phosphorylation (Fig. 1 and 2).

As requested, we performed ChIP-seq for the MED23 subunit in RAW264.7 cells. However, no significant signal enrichment was detected compared to the input (Figure R10. A), likely due to Mediator’s role as a co-activator lacking intrinsic DNA-binding activity and/or the absence of ChIP-grade Med23 antibodies. Nevertheless, we further performed ChIP-seq of Pol II and H3K27ac (Figure R10. B-D) in WT and Med23-defient RAW264.7 cells with or without VSV infection. Pol II ChIP-seq showed increased binding at inflammatory response genes that were upregulated in Med23$^{\Delta M}$ BMDMs after VSV infection, as identified in our RNA-seq analysis, despite that ChIP-seq of H3K27ac showed no clear difference. This supports a role for Med23 in regulating inflammatory gene expression at the transcriptional level (Figure R10. B and D).

Regarding whether the observed effects are due to Med23-mediated transcriptional regulation of RIG-I or an indirect effect via IFN-mediated induction, Reviewer #4 shared the same concern. We followed the suggestion of Reviewer #4 and investigated whether RIG-I expression is controlled by Med23 in cells lacking Ifnar1, with and without infection. We knocked down

Ifnar1 using siRNAs in wild-type and Med23 knockout RAW264.7 cells under both basal and viral-infected conditions and analyzed of RIG-I mRNA expression. The results revealed that in uninfected Ifnar1 knockdown cells, Med23 depletion significantly increased basal RIG-I mRNA levels, indicating IFN-independent regulation. Upon VSV infection, while wild-type cells showed expected RIG-I induction, Med23 knockout further enhanced RIG-I mRNA expression. Notably, Ifnar1 knockdown cells exhibited attenuated upregulation, yet Med23 depletion still significantly elevated RIG-I levels (Figure R10.F or revised Fig.5A). These finding strongly support that Med23 regulates RIG-I transcription primarily via an IFN-independent manner. Collectively, our data suggest that Med23 directly governs RIG-I transcription, which is likely independent of interferon feedback; whereas infection-induced ISG hyperactivation likely involves both direct (RIG-I-mediated) and indirect (IFN-mediated) mechanisms.

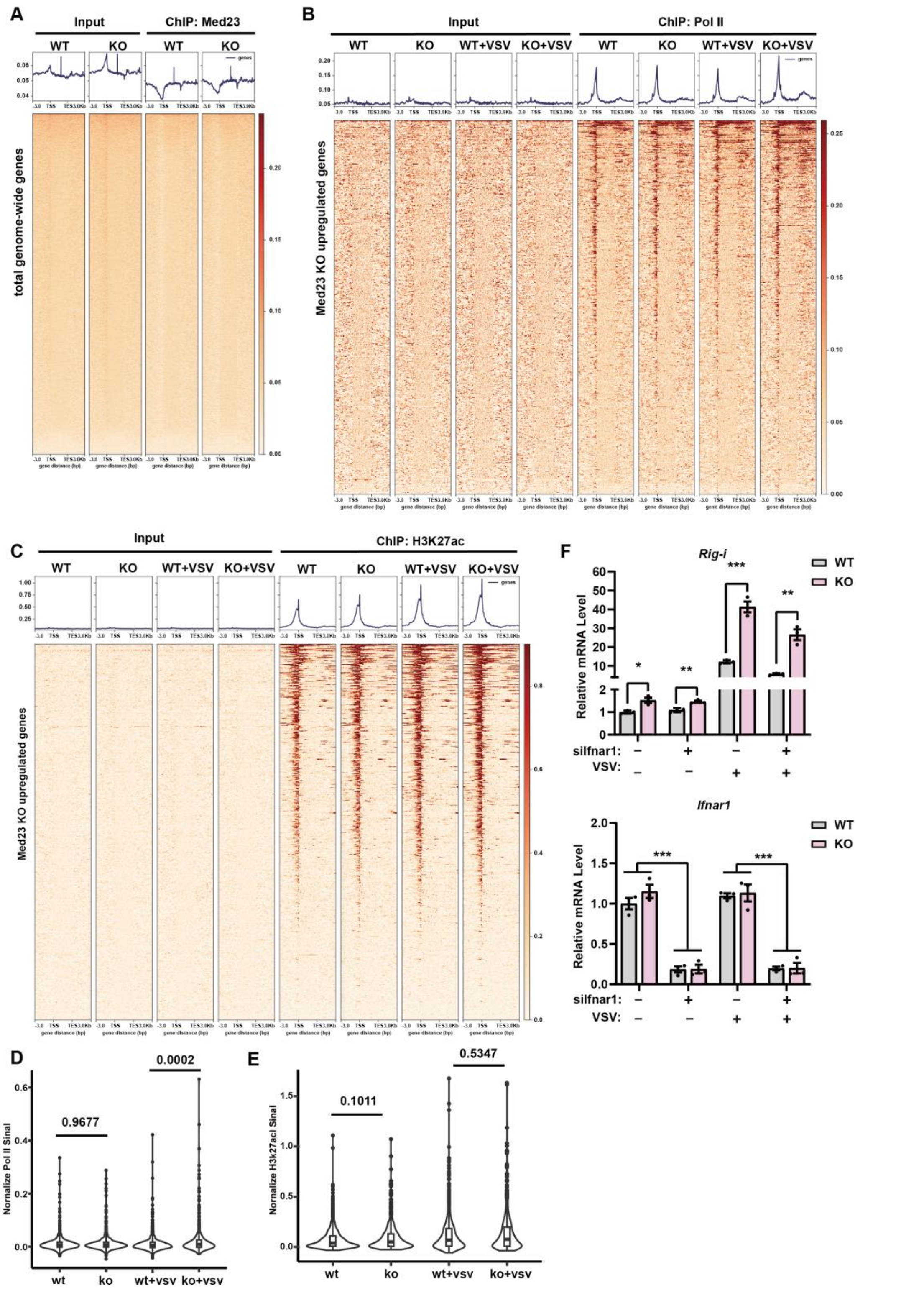

Figure R10. (A) Genome-wide occupancy of Med23 within the extended gene regions (-3 kb of TSS to +3 kb of TTS) in WT and Med23 knockout RAW264.7 cells. (B-C) ChIP-seq density heatmaps of Pol II (B) or H3K27Ac (C) in WT and Med23 knockout RAW264.7 cells, with or without VSV infection (MOI=0.5 for 6h), were shown within the extended gene regions (-3 kb of TSS to +3 kb of TTS) of genes upregulated in

Med23$^{\Delta M}$ BMDMs, as identified in our RNA-seq analysis under both basal and VSV-infected conditions. (D-E) Violin plot of the Pol II (D) or H3K27ac (E) binding intensity to genes upregulated in Med23$^{\Delta M}$ BMDMs, as identified in our RNA-seq analysis under both basal and VSV-infected conditions. (F) Expression of Rig-i and ifnar1 mRNAs in WT and Med23 knockout MEFs (n=3) with Ifnar1 knockdown (siRNA=50nM) infected with VSV (MOI=1). Statistical analysis was performed by two-tailed unpaired Student's t test; *$P < 0.05$, **$P < 0.01$ and ***$P < 0.001$.

Beyond the major defect in the overall model. There are multiple defects that impact the evaluation of this work:

(1) Figure 1A figure caption and actual panel are mismatched.

We sincerely apologize for this. We confirm that the Med23 Western blot data have now been added to the Fig. S1A.

(2) Not rigorous about statistical test and labeling of the significance. First, they did not mention the correction method used for multiple comparisons. Second, the labeling of the significance is arbitrary. For example, in figure 5c, non-significant is labeled for the upper panel but the label is missing for the lower panel siRIG-I #2. Third, some panels are presented with individual values, the others are not.

We have updated all figure legends to specify statistical tests and significance labeling. Individual data points are shown in all panels.

(3) Using qPCR as readout for Ifnb1 and interferon alpha, the name of the gene/transcripts are not labeled correctly. This is a big issue for interferon alpha, because there are 14 mouse interferon alpha subtypes.

The manuscript has been thoroughly revised to ensure that the qPCR data specifically measure Ifnα2.

(4) Line 146 says "immune escape of VSV" but the description of the viral load data started in the next paragraph.

We have revised the text: "These data suggest that Med23 controls the

immune response by regulating the activation of type I IFN signaling."

(5) Line 184 use "macrophage-specific" to describe LysMCre.

"Macrophage-specific" has been revised to "myeloid cell-specific" to describe LysM-Cre.

(6) Is there any immunopathology in vivo, due to increased interferon and inflammatory response in Med23$^{\Delta M}$ with and without viral infection?

As shown in Fig. 3E, in Med23$^{\Delta M}$ mice without viral infection, histological analysis of lung tissue revealed no significant differences in immunopathology compared to Med23$^{f/f}$ mice, while infected Med23$^{\Delta M}$ mice displayed reduced tissue damage compared to wild-type controls. Furthermore, we have now added new data in the revised manuscript, showing that Med23$^{\Delta M}$ mice exhibited less body weight loss upon low-dose VSV infection (revised Fig. 3F), and had significantly lower mortality than Med23$^{f/f}$ mice upon high-dose infection (revised Fig. 3G). Collectively, these results suggest that mice with myeloid-specific Med23 deficiency are protected from RNA virus infection. Nerveless, whether there is any immunopathology in the Med23$^{\Delta M}$ should be further examined in the future investigations.

(7) Line 262: Cxcl9, Cxcl10 are chemokines and Rsad2 is SAM family enzyme. Not cytokines.

This mistake has been corrected.

(8) Figure 4. It looks strange that the -log10(P) in D is close to 1 (P close to 0.1) Given C looks comparable with A, B and D should be in similar scale.

We sincerely appreciate the reviewer's careful attention to the statistical presentation in Figure 4D. However, we should point out that the Fig.4D is Gene Ontology analysis of significantly increased genes($P<0.05$) in BMDMs infected with VSV from Med23$^{\Delta M}$ mice compared with those from Med23$^{f/f}$ mice based on RNA-seq data. The raw P-value of the "cellular response to

chemokine" term is 0.032 (-Log10(P)=1.49485), instead of “P close to 0.1”.

(9) RIG-I is just one possible sensor. How about other viral sensors like cGAS, TLR3. Does Med23 specifically suppresses one sensor but not the other?

As shown in Figure R11, other innate immune sensors cGAS and TLR3 are not controlled by the presence or absence of Med23. Therefore, Med23 seems to specifically suppress RIG-I but not the cGAS and TLR3.

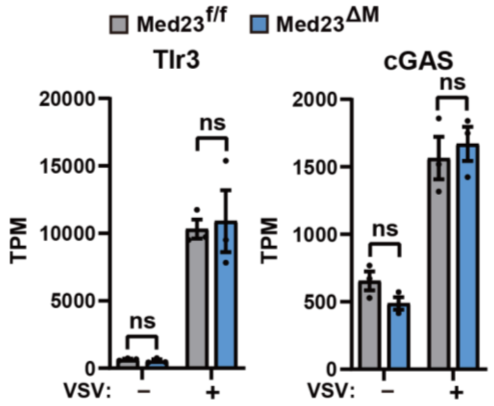

Figure R11. Analyze expression of Tlr3 and cGAS in our RNA-seq data of BMDMs.

(10) Figure 6F. The right panel, IP: HA, Foxo3 seems to have higher background in WT relative to other mediator or Cdk8 making the statement about the interaction less convincing.

In Figure 6F. The right panel, IP: HA, Foxo3 seems to have higher background in WT, but it is very important that Foxo3 signal in WT is much less compared to Foxo3 in HA-Med23 (the right lane). The Foxo3 background signal in WT may arise from non-specific binding associated with HA beads, which may be correlated with the higher input Foxo3 signal in WT. The weaker background signals for other Mediator subunits may reflect the minimal association of the Mediator complex with the residual co-IPed Foxo3 in WT.

Reviewer #4:

Innate immune sensors that induce type I IFNs play important protective roles in infection and cancer but may contribute to deleterious inflammation in other disease settings. As such, regulation of these sensors that include RIG-I is crucial. Tight control of RIG-I expression and activity by posttranslational modifications is well established and has been widely studied. Transcriptional control has received less attention but may be equally important.

By establishing that Med23 controls RIG-I gene transcription, this manuscript provides new and potentially highly relevant insights. Overall, the data are of high quality and support the conclusions well, and the manuscript is well written with nicely laid out figures. In my view, this manuscript is a good candidate for publication in PLoS Biology if the points below can be addressed by major revision.

My key concern relates to whether the observed effects are specifically due to Med23-mediated control of RIG-I transcription. Indirect effect, e.g. via IFN mediated induction of RIG-I transcription, seem possible, as are broader effects of Med23 on other pathways. Points 1-6 below include suggestions to address these issues.

We sincerely appreciate the positive assessment of our work. We have carried out a series of experiments based upon your suggestion. Below are our point-to-point responses.

Specific points

1. RIG-I protein expression appears increased in uninfected cells lacking Med23, e.g. Fig 5A (BMDMs), S4A/B (MEFs) or S4C (RAWs). Is this effect reproducible? Is RIG-I mRNA level increased in uninfected cells (e.g. in Fig 4A)?

Our data show that RIG-I protein levels are indeed elevated in uninfected Med23 KO cells across multiple cell types (BMDMs, MEFs, and RAW264.7 cells), as shown in Figures 5B, S4A-C; and these data are reproducible. RIG-I

mRNA levels are increased in MEFs (Fig. S4F-G), and exhibited a tendency to increase in BMDMs (Fig. 4A and Fig.5F) in uninfected cells. It is possible that the steady state culture conditions may trigger the basal level of differential expression of RIG-I, more or less affected by Med23. As the basal expression levels of RIG-I mRNA are rather minimal, we did not focus our investigation here. However, upon VSV infection, RIG-I mRNA levels are significantly elevated across multiple cell types. To determine which targets could be directly involved in the upregulation of the IFN response caused by Med23 deficiency, we observed that the presence or absence of Med23 did not alter IFNβ mRNA levels in response to MAVS, TBK1, or IRF3 overexpression, suggesting that Med23 targets an upstream component. Upon VSV infection, RIG-I expression was much higher in Med23 KO cells. The VSV-stimulated increase in the expression of IFNβ and IFNα2 in both WT and Med23-knockout cells was largely abrogated by RIG-I knockdown (Fig.5C). Moreover, RIG-I knockdown significantly blunted the Med23 knockout-induced increase in the phosphorylation of TBK1 and IRF3 (Fig. 5D). These results suggest that Med23-mediated suppression of innate immune responses against RNA viruses largely relies on RIG-I.

2. RIG-I is encoded by an ISG. Therefore, the authors should test if RIG-I expression is controlled by Med23 in cells lacking IFNAR, with and without infection.

To delineate the relationship between Med23 and interferon signaling in regulating RIG-I expression, we have conducted the suggested experiments using siRNA knocking-down IFNAR in WT or Med23 KO cells under both basal and viral-infected conditions (Figure R12 or Fig.S5A). In uninfected IFNAR1-KD cells, Med23 depletion still upregulate RIG-I mRNA levels, suggesting IFN -independent regulation. Upon virus infection, as expected, WT cells showed expected RIG-I induction, Med23 knockdown further enhanced RIG-I mRNA expression, and IFNAR1-KD cells exhibited slightly

attenuated upregulation. However, importantly, Med23 depletion is still able to significantly enhance RIG-I mRNA levels in the IFNAR1-KD cells upon VSV treatment, further suggesting the IFN-independent regulation of RIG-I expression by Med23.

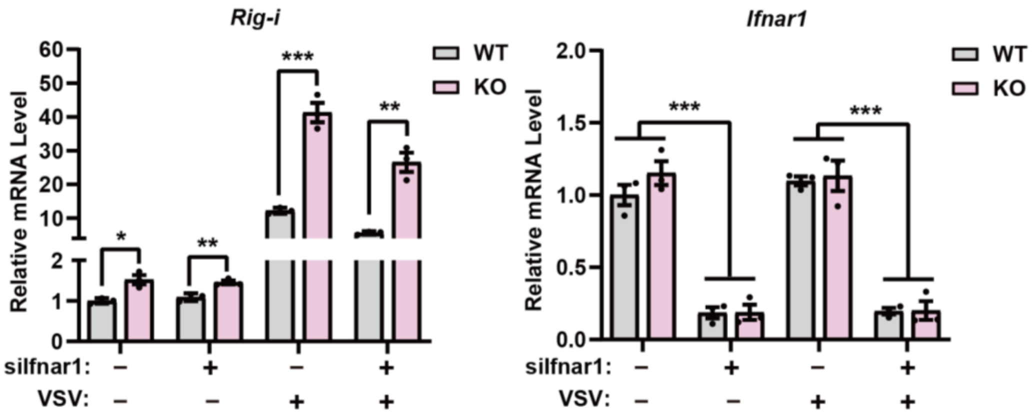

Figure R12. Expression of Rig-i and ifar1 mRNAs in WT and Med23 knockout MEFs (n=3) with Ifnar1 knockdown (siRNA=50nM) infected with VSV (MOI=1). Statistical analysis was performed by two-tailed unpaired Student's t test; *P＜0.05， **P＜0.01 and ***P＜0.001.

3. The RIG-I promoter analysis (Fig S5) would benefit from inclusion of the ISREs and other elements. Do these overlap with Foxo3 sites?

Per suggestion, ISREs have been included for the RIG-I promoter analysis (Fig. S5B). They do not overlap with Foxo3 sites.

4. To demonstrate specificity for RIG-I, the authors should induce IFNs in WT and Med23 KO cells using DNA transfection or with cGAMP, which both trigger the cGAS-STING pathway.

Following the reviewer's advice, we stimulated WT and Med23 KO cells with poly(dA:dT) (a synthetic dsRNA analog to activate the cGAS-STING pathway) and analyzed both IFN and RIG-I expression. Med23 KO cells exhibited comparable IFNβ, IFNα2, cGAS and RIG-I mRNA levels compared to WT cells after poly(A: T) transfection (Figure R13).

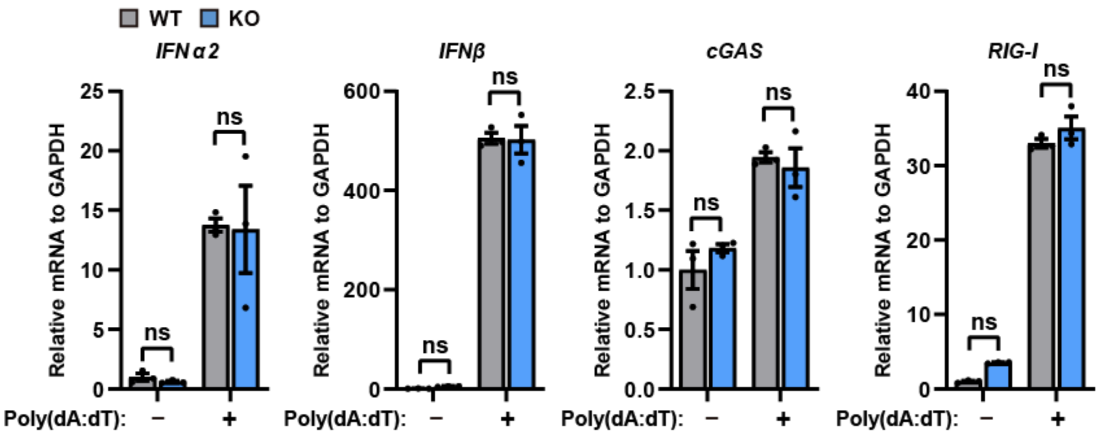

Figure R13. qPCR analysis of the levels of the indicated genes in Med23-knockout HeLa cells stimulated with poly (dA:dT) (10μg/mL, n=3) for 8h. Statistical analysis was performed by two-tailed unpaired Student's t test; ns, not significant.

5. Are other innate immune sensors controlled by Med23? Please analyse RNA-seq data in Fig 4 for other sensors, including MDA5 and LGP2.

As shown in Figure R14, other innate immune sensors MDA5 and LGP2 are hardly changed by the presence or absence of Med23.

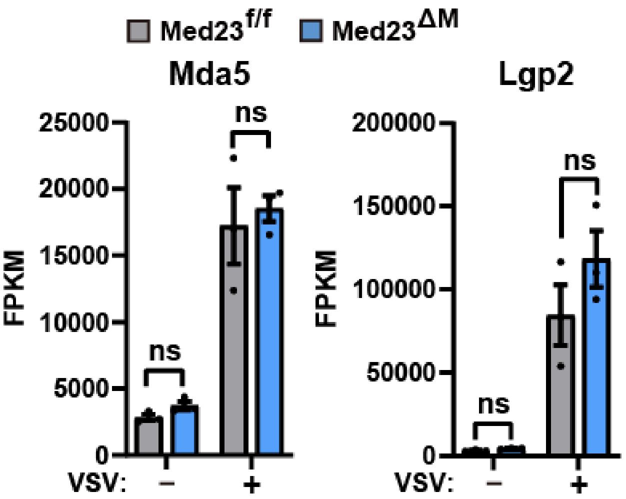

Figure R14. The expression of MDA5 and LGP2 in our RNA-seq data of BMDMs.

6. Fig 5A. Please include dose-titrations to ensure that the response is not maximal (which may mask differences between WT and KO).

To address reviewer's concern, we conducted the dose-titration experiment using expression plasmids for MAVS, TBK1, or IRF3 (0,100,200,300ng) in

both WT and Med23-KO cells. The results demonstrate that the presence or absence of Med23 had no significant effect on IFNβ mRNA levels during dose-dependent overexpression of MAVS, TBK1, or IRF3, further supporting the possibility that Med23 targets an upstream component of the RLR signaling pathway (Figure R15).

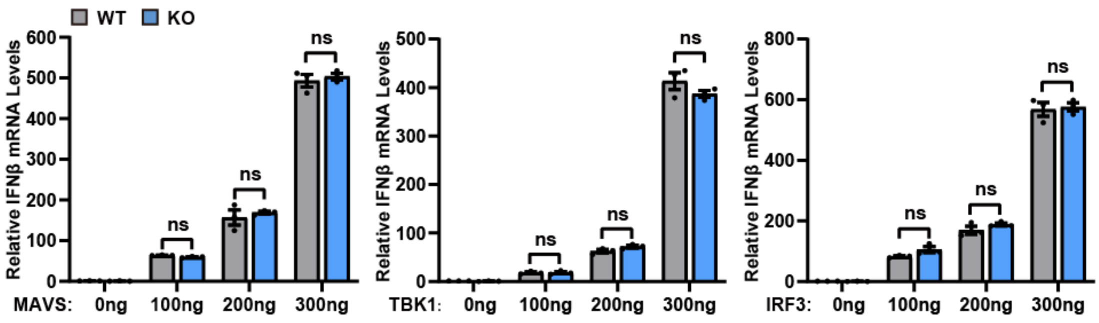

Figure R15. The expression of IFNβ mRNA in WT and Med23 knockout MEFs (n=3) transfected with increasing doses of expression plasmids for MAVS, TBK1 and IRF3 or empty control vector for 48 h. The results are presented relative to the expression of IFNβ mRNA in control cells. Statistical analysis was performed by two-tailed unpaired Student's t test; ns, not significant.

7. Fig 5D. Med23 protein levels appear to be increased in RIG-I-depleted cells. Is this reproducible? If yes, can the authors please comment on this apparent reciprocal regulation?

Our study demonstrates that Med23 plays a pivotal role in orchestrating antiviral immune responses. Specifically, the observed increase in Med23 protein levels in RIG-I-depleted cells raises the possibility of a reciprocal regulatory relationship between Med23 and RIG-I. While our current experiments cannot fully delineate the mechanistic basis of this interaction, we emphasize that this unresolved aspect does not conflict with the core finding that Med23-mediated suppression of innate immune responses against RNA viruses apparently relies on RIG-I. Future studies are needed to dissect the potential reciprocal regulation mechanisms linking Med23 and RIG-I activity.

8. Fig 5E. The authors may wish to consider measuring RIG-I mRNA decay in WT and KO cells after ActD treatment. If the authors' model is correct, mRNA

half-live is expected to be unchanged in KO cells.

Following the reviewer's advice, we performed Act D treatment in WT and Med23 KO cells to assess RIG-I mRNA decay rates. As shown in Figure R16 or revised Fig.S4E, RIG-I mRNA stability is unchanged in Med23 KO cells, supporting that Med23 regulates RIG-I at the transcriptional level.

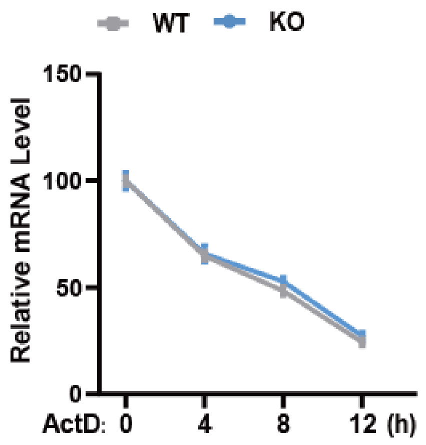

Figure R16. qPCR analysis of RIG-I in WT and Med23 knockout HeLa cells (n=3) treated with Act D (1mM/mL) for 0,4,8,12h.

9. Fig 5G-H. The RIG-I promoter luciferase reporter is not described in the methods and main text, and no citation is provided. Is this a new reagent generated in this study? If yes, please provide a detailed description and validation data for this reagent. Alternatively, if this is a published reagent, please provide a brief description and relevant citation(s).

The RIG-I promoter luciferase reporter used in Fig. 5G-H and Fig.6A is a new reagent generated in this study. The human or mouse RIG-I promoter region (2 kb upstream of TSS) was cloned into the pGL3-Basic vector upstream of the firefly luciferase gene. Specificity was validated by higher luciferase activity compared to the empty pGL3-Basic vector (Figure R17, human pGL3-RIG-I promotor; or Fig.6A, mouse pGL3-Rig-i promoter.). The dual luciferase reporter assay was carefully described in the revised Materials and Methods section.

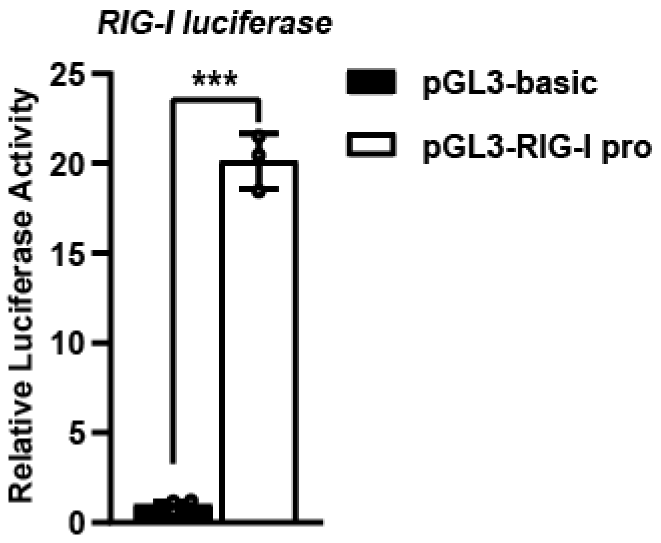

Figure R17. Luciferase reporter assays were conducted with HeLa cells transfected for 48 h with pGL3-RIG-I reporter plasmids and pGL3-basic control plasmids. Statistical analysis was performed by two-tailed unpaired Student's t test; **P < 0.01.

10. Figure legends should include information on the number of technical and biological repeat experiments for each panel, and whether pooled or representative data are shown. Bars and error bars need to be defined. Why do some graphs show individual data points (e.g. in Fig 5, panels F, G, H), whereas other graphs do not (e.g. in Fig 5, panels A, C, I)?

All figure legends have been revised to explicitly state the number of biological repeat experiments, and error bars were defined as mean ± SEM for each panel. And all graphs consistently show the individual data points now.

11. Many of the reviews cited in the introduction are outdated, incl. refs. 1, 3-6, 10, and 18-19. I recommend replacing with up-to-date reviews from the last few years.

Per the reviewer's suggestion, the Introduction has been updated to incorporate the most recent review articles.

12. Lines 109-111, 113-114, 120-124, 198-199, 201-202, 263-266, 319-321, 338. Please indicate in text that mRNA levels are measured here.

To clarify this point, we have revised the text to explicitly state that "mRNA levels" were measured.

13. Lines 160-161. The Med23 western blot mentioned as panel (A) in the legend is missing from the figure.

We confirm that the Med23 Western blot data have now been added to the Fig. S1A.

14. Line 222 "the fold change in" should be deleted as panel 3D shows relative expression, not fold change.

As suggested, the text has been revised by removing the phrase "fold change in" to align with the data presentation in Figure 3D.

15. Fig 3E. Can the authors quantify / score pathology in different sections from different animals to demonstrate reproducibility?

As requested, we have performed quantitative histopathological scoring on lung sections (Yang et al. 2022).The lung injure score was lower, along with reduced alveolar wall thickening, fibroblast proliferation, and extracellular matrix in Med23$^{\Delta M}$ mice compared to that in Med23$^{f/f}$ mice (Fig.3E).

16. Fig 4E and lines 294/295. Please clarify whether these data are with or without VSV.

The data in Fig. 4E were obtained without VSV infection. We have revised the text to explicitly state.

17. Line 273: use "ISGs" instead of "ISG genes".

We have revised to "ISGs".

18. Line 372: there is no panel S5C in Fig S5.

This has been corrected.

19. Fig 6D. Please include total TBK1 blot, and ideally also p-IRF3 and total

IRF3 blots.

Total TBK1, p-IRF3, and total IRF3 blots have been added to the revised Fig. 6D.

20. Line 431. "conditional" (not "conditioned")

This has been corrected as “conditional”.

Response references:

Akoulitchev, S., S. Chuikov and D. Reinberg (2000). "TFIIH is negatively regulated by cdk8-containing mediator complexes." Nature **407**(6800): 102-106.

Ding, N., C. Tomomori-Sato, S. Sato, R. C. Conaway, J. W. Conaway and T. G. Boyer (2009). "MED19 and MED26 Are Synergistic Functional Targets of the RE1 Silencing Transcription Factor in Epigenetic Silencing of Neuronal Gene Expression." Journal of Biological Chemistry **284**(5): 2648-2656.

Ding, N., H. Zhou, P. O. Esteve, H. G. Chin, S. Kim, X. Xu, S. M. Joseph, M. J. Friez, C. E. Schwartz, S. Pradhan and T. G. Boyer (2008). "Mediator links epigenetic silencing of neuronal gene expression with x-linked mental retardation." Molecular Cell **31**(3): 347-359.

Litvak, V., A. V. Ratushny, A. E. Lampano, F. Schmitz, A. C. Huang, A. Raman, A. G. Rust, A. Bergthaler, J. D. Aitchison and A. Aderem (2012). "A FOXO3-IRF7 gene regulatory circuit limits inflammatory sequelae of antiviral responses." Nature **490**(7420): 421-+.

Liu, X., L. Cui, Y. Tao, S. Xia, J. Hou, X. Cao and S. Xu (2024). "The deubiquitinase BAP1 and E3 ligase UBE3C sequentially target IRF3 to activate and resolve the antiviral innate immune response." Cell Reports **43**(8).

Liu, Z., X. Yao, G. Yan, Y. Xu, J. Yan, W. Zou and G. Wang (2016). "Mediator MED23 cooperates with RUNX2 to drive osteoblast differentiation and bone development." Nature Communications **7**(1).

Mun, S. H., P. S. U. Park and K. H. Park-Min (2020). "The M-CSF receptor in osteoclasts and beyond." Experimental and Molecular Medicine **52**(8): 1239-1254.

Ray, A. and B. N. Dittel (2010). "Isolation of Mouse Peritoneal Cavity Cells." Journal of Visualized Experiments(35).

Ren, X. X., D. C. Wang, G. R. Zhang, T. Y. Zhou, Z. Wei, Y. Yang, Y. J. Zheng, X. Q. Lei, W. Y. Tao, A. M. Wang, M. S. Li, R. A. Flavell and S. Zhu (2023).

"Nucleic DHX9 cooperates with STAT1 to transcribe interferon-stimulated genes." Science Advances **9**(5).

Soutourina, J. (2018). "Transcription regulation by the Mediator complex." Nature Reviews Molecular Cell Biology **19**(4): 262-274.

Wang, Z. C., D. Cao, C. H. Li, L. H. Min and G. Wang (2019). "Mediator MED23 regulates inflammatory responses and liver fibrosis." Plos Biology **17**(12).

Xu, C., H. Kleinschmidt, J. Yang, E. M. Leith, J. Johnson, S. Tan, S. Mahony and L. Bai (2024). "Systematic dissection of sequence features affecting binding specificity of a pioneer factor reveals binding synergy between FOXA1 and AP-1." Molecular Cell **84**(15): 2838-2855.e2810.

Yang, L. P., S. C. Chen, Q. Zhao, C. H. Pan, L. A. Peng, Y. Han, L. L. Li, J. Y. Ruan, J. Y. Xia, H. Yang, F. Xu and G. H. Cheng (2022). "Histone deacetylase 3 contributes to the antiviral innate immunity of macrophages by interacting with FOXK1 to regulate STAT1/2 transcription." Cell Reports **38**(4).
